# Microsatellite markers-aided dissection of iron, zinc and cadmium accumulation potential in *Triticum aestivum*

**DOI:** 10.7717/peerj.15229

**Published:** 2023-04-17

**Authors:** Asima Rasheed, Javed Ahmad, Majid Nadeem, Muhammad Abdul Rehman Rashid, Farrukh Azeem

**Affiliations:** 1 Department of Bioinformatics and Biotechnology, Government College University Faisalabad, Faisalabad, Punjab, Pakistan; 2 Wheat Research Institute, Ayub Agricultural Research Institute (AARI), Faisalabad, Punjab, Pakistan

**Keywords:** Wheat, Biofortification, Iron, Zinc, Cadmium, SSR markers, Marker assisted selection, Bioavailability, Gene specific primers, Tassel

## Abstract

**Background:**

Wheat is a staple cereal food around the globe. It provides a significant source of proteins, carbohydrates, and other micronutrients to humans. When grown on cadmium (Cd) contaminated soils, the uptake of trace elements *e.g*., iron (Fe) and zinc (Zn) has also been affected drastically that in turn affected the wheat grain.

**Methods:**

In this study, wheat accessions were used to investigate the impact of soil application of Zn (5 mg/kg, 20 mg/kg) and Cd (0 mg/kg, 10 mg/kg) on accumulation of these elements in wheat grains. A total of 45 Fe, Zn, and Cd transporter-related genes were used to design 101 gene-specific SSR (simple sequence repeat) markers.

**Results:**

In response to Cd stress, application of 20 mg/Kg Zn improved Fe (64.6 ug/g) and Zn (48.3 ug/g) accumulation in wheat grains as well as agronomic traits. Marker trait association revealed that SSR markers based on *NAM-B1* gene (PR01 and PR02) were associated with Zn accumulation. Similarly, SSR markers based on *TaVTL5-2B_5* (PR19 PR20), *TaVTL5-2B_2* (PR25, PR26), *TaVTL5-2D_3* (PR30), *TaVTL2-2A* (PR31), *TaVTL1-6A* (PR32), and *TaVTL2-2D_1* (PR37) were significantly associated with Fe accumulation, while HMA3-5B1 (PR62) and *TaNRAMP3-7D* (PR89) were linked to Cd accumulation in grains. The highly associated markers may be used in marker-assisted selection of suitable wheat genotypes for breeding bio-fortified varieties with low Cd accumulation.

## Introduction

Soil serves as the major and natural home of essential nutrients required by plants for proper functioning and development. At the same time, it also contains a trace amount of heavy metals but can become contaminated with higher concentrations of these metals through human activities (Rizwan et al., 2016a). Agricultural soil with heavy metal contamination *e.g*., Cadmium (Cd), chromium (Cr), lead (Pb), arsenic (As), or mercury (Hg) puts both ecosystem and people at risk and compromises the safety of food supply, lowers the food quality by reducing nutrient uptake, and makes it more difficult to use land for agricultural production. Collectively, all these factors have negative impacts on future global food security (González Henao & Ghneim-Herrera, 2021). Cadmium (Cd), one of the heavy metals, has drawn a lot of attention and is the principal barrier to food safety and the quality of agricultural land (Arshad et al., 2016; Genchi et al., 2020; Sardar, Ahmed & Yasin, 2022). Although Cd has no necessary functions in plant growth, it has been found to accumulate at a higher rate in plant systems through roots. On the other hand, Zn transportation pathways also serve as one of the main routes for Cd to enter the plant system, as Cd is an analog of Zn metal (Clemens, Palmgren & Krämer, 2002). Furthermore, industrial and sewage wastes and fertilizer applications along with natural processes also play role in the deposition of Cd into the soil environment (Rizwan et al., 2016b). It is readily taken up by crops and transferred to edible parts where it can be accumulated to a relatively high concentration. For example, 620 µg/kg Cd has been reported in wheat grains (Li et al., 2011). The accumulation of Cd also promotes the production of reactive oxygen species (ROS), which can have a harmful impact on the total protein content in plants. As a result, the plant’s defense system is activated to overcome the hazardous effects of Cd (Sardar et al., 2022).

Wheat (*Triticum aestivum*) is one of the most cultivated crops worldwide and is consumed as a principal ingredient of food. It provides essential minerals and vitamins required for metabolism maintenance and functioning (Akhtar et al., 2013; Igrejas & Branlard, 2020). The Green Revolution has led to significant improvements in yield and tolerance to harsh environmental conditions in plants. However, the focus on crop yield has resulted in a neglect of nutritional traits, leading to a lack of Fe and Zn in edible portion of many cereals, including wheat. Every year more than five million deaths are reported to Fe/Zn malnutrition (Sharma et al., 2020). The standard range of Fe and Zn accumulation in wheat grains is 29–73 mg/kg and 7–85 mg/kg (Bassi et al., 2021) respectively. Whereas, their deficiencies constrain grain production as well as the nutritional quality of grain. Furthermore, a considerable amount of dietary fibers, starch, and nutrients are lost during the milling process used to form refined white flour, which is also linked to Fe/Zn deficiencies in the human diet (Thielecke, Lecerf & Nugent, 2021). These circumstances require the development of a prompt solution to mitigate the nutritional gaps in wheat and to reduce hidden hunger (Chattha et al., 2017; Rizwan et al., 2019). The supply of Fe and Zn content in the wheat crop can be enhanced either by raising their bioavailability or by elevating their level in edible parts through biofortification strategies (Wakeel et al., 2018). These two options strongly depend upon the calcium concentration of the soil. Although wheat has anti-carcinogenic and antioxidant properties, it also contains phytic acids that function as anti-nutrients. The main antinutrients involved in reduction of mineral bioavailability in food are phytates, tannic acid, and polyphenols. About 60% of the minerals and 85% of the phytic acid in the wheat kernel are localized in the aleurone layer. Phytic acid, also known as myo-inositol hexaphosphate (InsP6) or phytate, is a major inhibitor of Fe and Zn absorption (Aiqing et al., 2022). Electrostatic interactions of phytic acid with proteins and minerals produce insoluble complexes that decrease phytase activity in intestines. As a result, digestion and bioavailability are reduced in the gastrointestinal tract of humans (Akhter et al., 2012). In Pakistan, phytate concentrations in edible parts of wheat and rice have been reduced to increase the bioavailability of Fe/Zn. But high fiber or tannin are also major elements involved to limit the bioavailability of Fe and Zn (Lestienne et al., 2005). Improving the bio-absorption of minerals is one of the most effective methods to lower the amount of phytic acid in grains. The primary risk associated with this approach is that the properties of phytase such as its ability to reduce oxidative stress and protect body from colon cancer and other malignancies may also be affected (Vucenik & Shamsuddin, 2003; Lemmens et al., 2018). Therefore, the breakdown of phytate in grains could greatly raise cancer susceptibility, making this method discouraged.

The present study aimed to analyze a range of wheat genotypes for Fe, Zn, and Cd accumulation in grains and identify gene-specific SSR markers linked to these metals. The bioavailability of Fe and Zn was also evaluated by their digestibility under simulated physiological conditions. TASSEL was then used to evaluate the association between Fe/Zn accumulation with primers. To the best of our knowledge, this would be the first study in Pakistan that reported wheat grains with reduced Cd accumulation while increasing Fe/Zn levels.

## Materials and Methods

### Collection of plant material

The seeds of 189 wheat accessions were collected from the Wheat Research Institute, Ayyub Agriculture Research Institute (AARI), Faisalabad, Pakistan. Accessions FA-01 to FA-68 and FA-70 to FA-120 represented germplasm obtained from AARI. While accession FA-69 represented ZINCOL-16 and FA-121 to FA-189 represented approved wheat varieties (Data S1).

### Experiment I: phenotyping wheat accessions for agronomic traits in pots

The experiment was conducted at the Botanical Garden of Government College University, Faisalabad, Pakistan, using plastic pots (40 cm × 25 cm) filled with 8 kg of soil. To avoid fungal or microbial population, the seeds were sterilized by shaking them in 5% sodium hypochlorite (NaOCL) for 1–2 min and then rinsing them with sterile water. After sterilization, five seeds were sown in each pot and two levels of Zn (5 mg/kg, 20 mg/kg) and Cd (0 mg/kg, 10 mg/kg) were applied under factorial design along with a control group. The entire experiment was designed in triplicates using a completely randomized design (CRD) arrangement. After 14 weeks, data of different agronomic traits including plant height, spike length, number of grains/spike, days of flowering, number of tillers/plant, spike length, and 1,000 grain weight were evaluated using standard procedures. The atomic absorption spectrophotometer was used to measure Fe, Zn, and Cd contents in ground seeds as described by Zorrig et al. (2010).

### Experiment II: Markers selection, validation, and genotyping of material

About 45 genes associated with Fe, Zn, and Cd homeostasis (Data S2) were selected from the previously published literature on different crops (Clemens, Palmgren & Krämer, 2002; Palmgren et al., 2008). A total of 45 SSR primers were found and validated from the flanking region of these genes through NCBI primer blast (https://www.ncbi.nlm.nih.gov/tools/primer-blast/). At the seedling stage, fresh leaves from each genotype were harvested and the whole genomic DNA of each genotype was extracted using the cetyltrimethylammonium bromide (CTAB) technique, as described by Murray & Thompson (1980). The extracted genomic DNA, along with selective material was used for the polymerase chain reaction (PCR), and the resulting amplicons were separated using agarose gel electrophoresis. The linkage of SSR markers and the accumulation of Fe, Zn, and Cd in wheat grains was evaluated using genotyping and phenotypic data. The markers-traits association was discovered using a mixed linear model regression technique in TASSEL, as described by Bradbury et al. (2007).

### Fe and Zn bioavailability study

#### Total mineral content

The total Fe and Zn content of wheat flour was determined by nitric-hydrochloric acid digestion. Nitric acid (5 ml) was added to wheat flour (1 g) in a 250 ml digestive tube and kept in the fume hood overnight. After 24 h, freshly prepared aqua regia (three parts of 37% HCl and one part of 65% HNO_3_) was added to the tube and heated at 200 °C for 45 min or until the volume reduced to about 1 ml and the fumes disappeared. After cooling, interior tube walls were washed with distilled water to avert the loss of sample and filtered with Whatman filter paper. In the end, a sufficient amount of distilled water was added to make the final volume (50 ml). An atomic absorption spectrometer (novAA® 350; Analytik Jena, Neckarsulm, Germany) was used to quantify the concentration of metals in the digestion mixture.

#### In vitro gastrointestinal digestion

Grain samples of 189 wheat accessions were used to evaluate Fe/Zn bioavailability by utilizing *in vitro* gastrointestinal digestion method described by Akhter et al. (2012) with apt modifications. Briefly, 3 g wheat flour was homogenized in 120 ml distilled water. The pH of the samples was maintained at 2.0 with 6 M/L HCL. The pH was checked and readjusted to 2 every 15 min. Subsequently, pepsin solution (1 ml) was added to the mixture followed by incubation in a shaking incubator (37 °C, 120 revolutions/min) for 2 h. An aliquot of gastric digest (12.5 ml) was obtained and raised to 120 ml with deionized water followed by the addition of pancreatin bile extract and pH was adjusted to 7.5 by 0.5 M NaOH. Afterward, 25 ml of 0.2 M NaHCO_3_ was added and segments of dialysis tubing (Molecular mass cut off 10 kDa) were positioned on the flask containing gastric digest. It was then incubated in a shaker at 37 °C for 30 min. Later on, pancreatin-bile extract (5 ml) was added and incubated for 2 h until its pH reached 7. Bioavailability of Zn and Fe in the dialyzate was evaluated by atomic absorption spectrometry and calculated by the following formula:

${Bioavailability\; }\left( {\rm \% } \right) ={\; }\displaystyle{{Y} \over {Z}}{\; } \times 100$

where Y and Z represent the element content of the bioavailable fraction (mg mineral element/100 g grain) and the total Zn or Fe content (mg mineral element/100 g grain) respectively.

### Population structure

The population structure of 189 wheat genotypes was analyzed using structure v2.4.2 software (Pritchard, Stephens & Donnelly, 2000) by utilizing 101 alleles of 45 SSR primer pairs. Ten independent simulations from one to 10 were allocated to each population number (K) with 10,000 repetitions and 50,000 MCMC (Markov Chain Monte Carlo) replication under related frequency and admixture models. The Structure Harvester tool (Earl & VonHoldt, 2012) was employed with Evanno’s 1K method to obtain optimum K and LnP(D) values (Evanno, Regnaut & Goudet, 2005). The population kinship and PCA analysis were performed in the R-based statistical tool GAPIT v3. (Lipka et al., 2012).

### Phylogenetic analysis

The phylogenetic analysis was carried out to identify the pedigree-based relation among genotypes. Cluster analysis of germplasm was carried out with DARwin ver. 6 software (Perrier & Jacquemoud-Collet, 2006) that used the neighbor-joining (NJ) method to calculate the Nei genetic distance among genotypes (Nei, 1978). The tree was updated and visualized using the Evloview web tool (He et al., 2016).

### Experiment III: marker traits association

The targeted-genes-based markers-traits association analysis was performed in JAVA environment using trait analysis by association, evolution and linkage (TASSEL) program v 4.0. The population PCs based general linear model and the mixed linear models were used to evaluate the markers’ traits association. The genotypic data were taken as the fixed variant against the phenotypes, and population structure (K-matrix and PCAs) as random variants. The association analysis was subjected to 1,000 permutations for results validation. The results for various stress levels and treatments were further evaluated manually in MS excel.

## Results

### Experiment I: phenotypic evaluation

#### Phenotyping wheat accessions for agronomic traits

The Zn and Cd contents in wheat grains were not related to each other, but they were both negatively correlated with grain yield. Cd stress reduced plant height, 1,000 grain weight, number of tillers per plant, spike length, and number of grains per spike as compared to control conditions. The application of Zn enhanced the morphological and yield traits of wheat. Among two levels of Zn application, the high level (20 mg) had the maximum and more significant improving effect on agronomic traits, followed by the low level (5 mg) of Zn (Table 1 and Data S3). The combination of 10 mg Cd with 20 and 5 mg Zn showed improved patterns as compared to Cd-stressed plants. A combination of 20 mg Zn application and induced Cd stress recorded greater plant height, 1,000 grain weight, number of tillers per plant, spike length, and number of grains per spike, while the least was observed for wheat plants grown under 5 mg Zn and Cd stressed condition (Table 1). A study conducted by Farooq et al. (2020) also observed a higher impact of Cd on wheat grown at low levels of Zn. The present study proved that Zn supplements in soil fertilizers could reduce the effects of Cd and improve the agronomically important traits of wheat plants. During the early stages of the crop, a higher level of Zn enhances the dry matter and root growth, making it a fundamental micronutrient for seed germination.

**Table 1:** Phenotypic evaluation of collected wheat accessions against various treatments.

	Controlled conditions			Cd10			Zn5			Zn20			Cd10+Zn5			Cd10+Zn20		
Trait	Min	Max	Ave.	Min	Max	Ave.	Min	Max	Ave.	Min	Max	Ave.	Min	Max	Ave.	Min	Max	Ave.
Plant height (cm)	60	110	99.4	45.67	85.86	65.5	58.2	112.4	105.5	69.1	115.3	108.2	55.6	95.5	80.1	54.5	98.1	85.6
Days to 50% heading	98	109	102.69	80	98	92.3	96	108	103.5	97	106	98.2	84	100	97.2	99	108	103.3
Number of tillers per plant	2.56	7.5	4.6	1	5.6	3.2	2.9	8.4	5.1	2.8	9.1	5.2	1.6	5.9	4.1	2.1	6.1	4.4
Spike length (cm)	3.9	14.2	7.9	2.9	12.1	6.32	3.7	16.1	8.6	4.1	15.1	8.4	3.1	14.1	6.9	4.2	13.9	7.6
Number of grains per spike	5	74	36.34	6	88	26.1	8	78	38.2	7	67	37.2	7	82	30.1	8	81	32.2
1,000 grain weight (g)	22.4	54.3	39.1	20.8	46.7	28.9	24.3	56.1	38.3	25.2	56.4	38.9	21.63	51.1	29.6	20.1	49.2	30.1
Iron (ug/g)	6.9	382.3	53.3	2.1	274	22.6	3.05	535.2	59.3	3.92	642.9	64.6	2.9	425.7	30.3	3.5	510.9	32.2
Zn (ug/g)	4.5	70	33.3	6.5	48.9	27.3	9.9	75.3	40	12.4	82.3	48.3	7.9	55.4	29.4	8.9	58.9	32.5
Cd (ug/g)	0	0	0	0.91	72.13	26.95	0	0	0	0	0	0	0.9	65.9	23.9	0.2	54.6	18.9

#### Traits based germplasm evaluation

**Figure 1:**
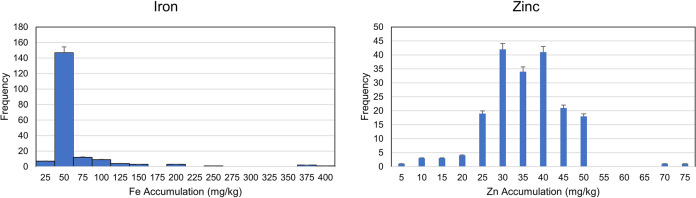
Frequency distribution for zinc and Iron accumulation in diverse population of 189 wheat genotypes.

A wider range of phenotypes (Fe and Zn accumulation) was detected in the genotypes. The analysis of variation (ANOVA) was performed to reveal the genetic and environmental variations. A highly significant variation among genotypes indicated the availability of genetic variation among the selected genotypes, while a relatively non-significant environmental variation also indicated the accuracy of pot experiment (Table 1 and Data S3). The trait frequency distribution of Fe and Zn accumulation was performed and a normal distribution was observed among the genotypes for Zn accumulation. On the other hand, the frequency distribution for Fe accumulation was highly skewed and concentrated within a range of 25 to 75 mg/kg (Fig. 1 and Data S3). Further coefficient of variation (CV) was estimated. The CV also indicated a higher degree of variability in the selected germplasm (Descriptive). The genetic and environmental variations were used to estimate the possible heritability of genotypes for any of the next breeding programs.

#### Fe, Zn, and Cd contents in wheat grains

The wheat grains were evaluated for their available micronutrients. Reduced concentrations of Fe and Zn were observed in wheat grains that were raised from Cd-contaminated soil to control. Cd was transferred from roots to shoots and eventually to grains. It caused a reduction in the accumulation of micronutrients. Sole application of Zn enhanced Fe and Zn concentrations in grains of all wheat plants (Table 1). Among two levels (5 and 20 mg/Kg) of Zn, the higher concentration showed maximum Fe and Zn accumulation in grains compared to the lower Zn concentration. This may be due to sufficient Zn supply, which enhances germination and seedling growth owing to high cell division and expansion, enzyme activity, and protein synthesis. When 5, 20 mg/kg Zn was applied in soil contaminated with Cd (10 mg/kg), the Fe and Zn concentrations in wheat grains declined. However, the concentrations were still higher compared to the grains treated only with Cd.

### Experiment II: genotypic evaluation

#### Selection of markers and genotyping

The genes linked to the mineral accumulation were reviewed and the genes for Zn, Fe, and Cd were selected for further analysis. The genomic information for selected genes (5 kb $\pm$ genomic regions of a gene) was used to design primers as markers for Zn (18 primer sets), Fe (27 primer sets), and Cd (56 primer sets). All the designed primer sequences were verified against online databases (NCBI and Ensembl plants). The non-specific primers were removed from consideration (Table 2, Data S4). The 45 primer pairs were used to genotype the 189 wheat accessions which yielded a total of 101 alleles for further evaluation.

**Table 2:** List of primers used to identify/mark the genes related to Zn, Fe, and Cd in wheat genome.

Gene name	Forward primer sequence	Reverse primer sequence
Zn		
bZIP28	ACTGATCAGCAATGTCAACTT	GCTCAACATTCTGCTTAGAAA
bZIP23	GAGACAGCGAGTTATTGGTT	TTCAAACTCAAGTAGTGGATGA
bZIP22	GACGACAGCGATGATGAC	GAATAACGCGCAGAGAGA
bZIP21	ATGCATGTGTCAGGTAGAGAA	TTTGCACTTTTCTCTGGTAAG
bZIP20	TGTGCATTTAAGTACAGGAAAA	CGACTTGTACTTCCAGTGAAT
bZIP1-D	AACTGAGGCTTAGATGAATCC	CAGTACAGGGCTGACTTTTT
bZIP1-A	GGAGGACGTCTAATCTCATTT	CATCTTCGTCCTCCTCATC
bZIP19	TATGGGAATTGTCACAGTTTT	CAAGTAGTAGCACCTTTGCAT
bZIP1	TACCACCAAGGTAAAAAGACA	GAGGAAAATTCTCAGTCTGTGT
bNAM-B1	GCTTTCATGAGAAATACAACC	AAAAACAGAAAAACTGGCAAC
Fe		
bVIT1-2D	GCTCAATTCTGTCTGTTGC	GGACACGCCCATGATGAC
bVTL4-4D	TCCATTAGGAGTAGAGATGACC	TATATAGCGACGAGGCAGTAG
bVTL5-2B_5	GCCGAAATGATAACGAAATA	ACAATTGCTAGCGATAATGTG
bVTL5-2D_3	TGGAATGGATGGGTTAATAAT	GTGTGGCTAGTGCGTACAT
bVTL2-2A	TCGACTCGACAATTTCTCTAA	TCACTTGTGTATTCCCCTAAA
bVTL1-6A	GAAGGGAGGAGTAGCAGTAGA	GCCTTGAGGTGGAACTGC
bVTL5-2B_2	TAGTACTACCATGGCCGAAA	TACAATGGCTAGCGATAATGT
bVTL4-4A	GCTTCCAATGCCTATATTTCT	TTTTAGAGCATCTTCAACAGG
bVTL5-2B_6	GGATGGGTTAATAATCTAGGC	ACTGTGTGGCTAGTGCGTA
bVTL5-2B_1	TACCATGACCGAAATGATAAC	TACAATGGCTAGCGATAATGT
bVIT2-5D	CAGGTCACTCTCTCACTTGAC	CCCGTACTCAGAGAGTATGTC
bVTL1-6B	GTGGTGGACGTGTACCTG	CTTGTACGCCCTGTTGAC
bVTL4-4B	GTGGTTGGGTAGCTCGTG	AGAGAGACGCCTTTTTCTATC
bVTL2-2D_1	CTCCTTCTTGAGTAGCTCGAT	GCGGGCCATGTAGTTGAC
bVTL5-2B_4	AAATTGTCGCTGTTTCACATA	TACAGTTCAAGAACCTTCCAG
Cd		
bHMA2-7A1	AAATTCTAGCCCATTTAACCA	TGACTTCAGTTTGGATGGTAT
bHMA2-7B1	GGTCCAAGTTTCTATTCACAGT	AACTGCATGAATCTAACAGGA
bHMA2-7D1	GTAATAGCCCGTGGATGTAG	ATTAACTGGCAAACCCACCTA
bHMA3-5A1	GGTGATGGTTGTTAAATTTGT	GAATGTCATGTTTCGAGCTAT
bHMA3-5B1	AGAGGACAGGGCAGAGTG	AGTTAACCCGATTTCTGACA
bHMA3-5D1	ATGATGACATGTTGGTTGTTT	AGTTTTCCACTTCTCCTCATC
bNRAMP1-7A	TGTATAATGGCCTTGGTTATC	GTAGTGCATGTGCTTCCTTAC
bNRAMP1-7B	CTCTCATCCCTCTTCTCAAGT	TAGGTACATGGATGGATTCAG
bNRAMP1-7D	ATACTGTCATTTGAGCTACCG	CTTCCACAGCCAAAAATATAA
bNRAMP2-4A	AAGATAGAGCAGGTTTTCACC	GTTAGGTGGTGTCGAGGTC
bNRAMP2-4B	CCCTCTTCTCTTCCAAATAAC	GTTTGTTCCTCTTTTTCTTGG
bNRAMP2-4D	AAGGTCTCCATCTCCATCTC	CCGATGCACATGAGGAAG
bNRAMP3-7A	GAAAGAGGGAGATAGACGAAA	AGAAAGGCGAAACCTAAGTC
bNRAMP3-7B	GGAGAAGACGAGGTAAGAAGA	GAGACCGGAGAGAGAGGA
bNRAMP3-7D	TTATTGTTATTTGGAGGGTAGG	TAAACTTTAGGGCCTTTATCC
bNRAMP4-U1	CCCTCTAGCAGATCTCGTC	GTAGGAACCTTCTTGTGCTTC
bNRAMP5-4B	ATGATACTGTCGTTTGAGCTG	GAGTACGTGTCCAAAAACAAG
bNRAMP5-4A	CTCCCTCACTGTCAATATCAG	TCGTGCGAAATATAGTAGTGG
bNRAMP5-4D	ATTAGGGAGAACGAAAATGAC	CTGGCTGCTAGTAAGTCGTAG

#### Population structure

**Figure 2:**
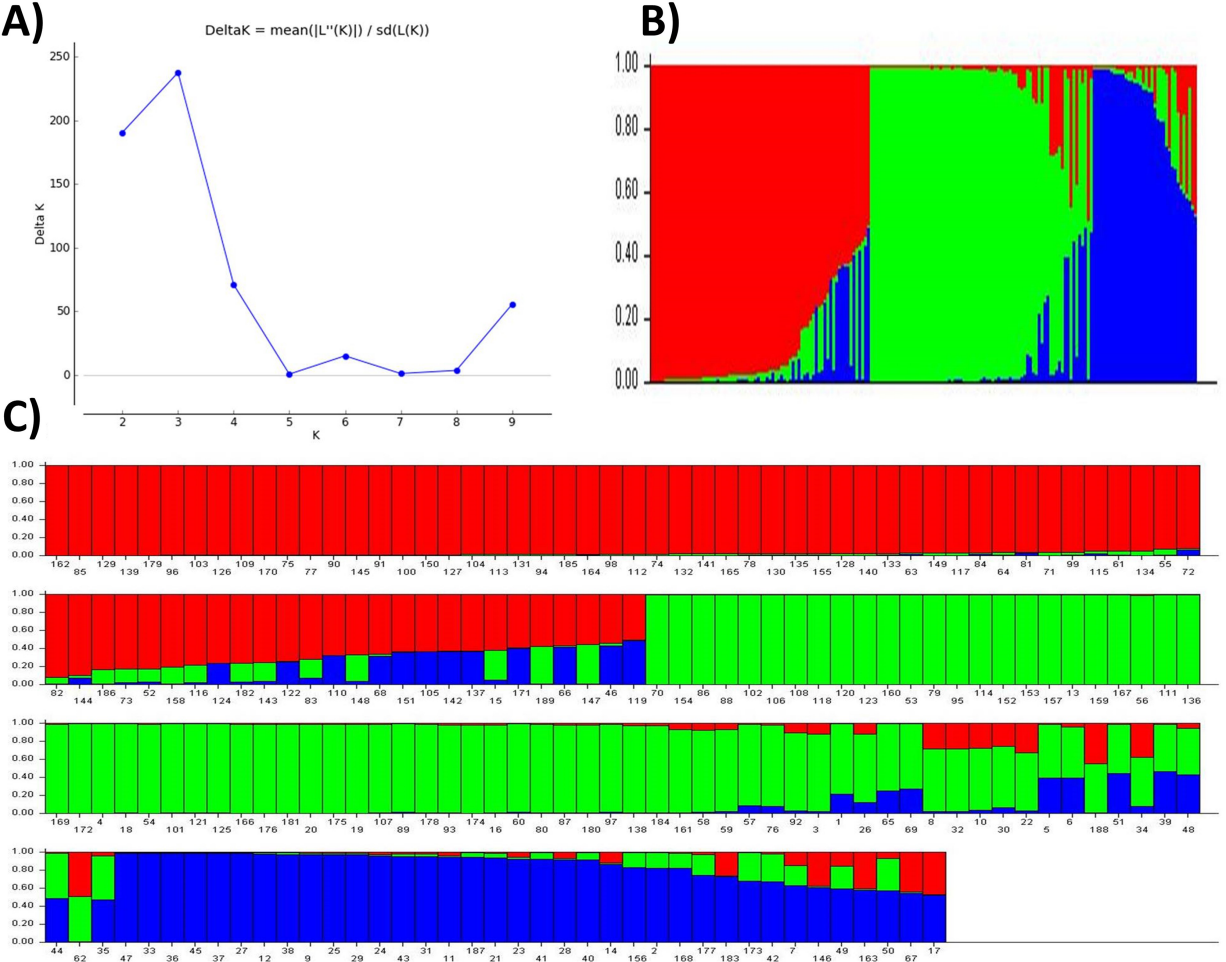
Population structure and admixture analysis of 189 wheat genotypes revealed by 101 SSR markers. (A) The maximum delta K based of LnP(D) Scores, (B) three subpopulations, (C) individual scores for candidates in three subpopulations indicating their degree of admixture.

**Figure 3:**
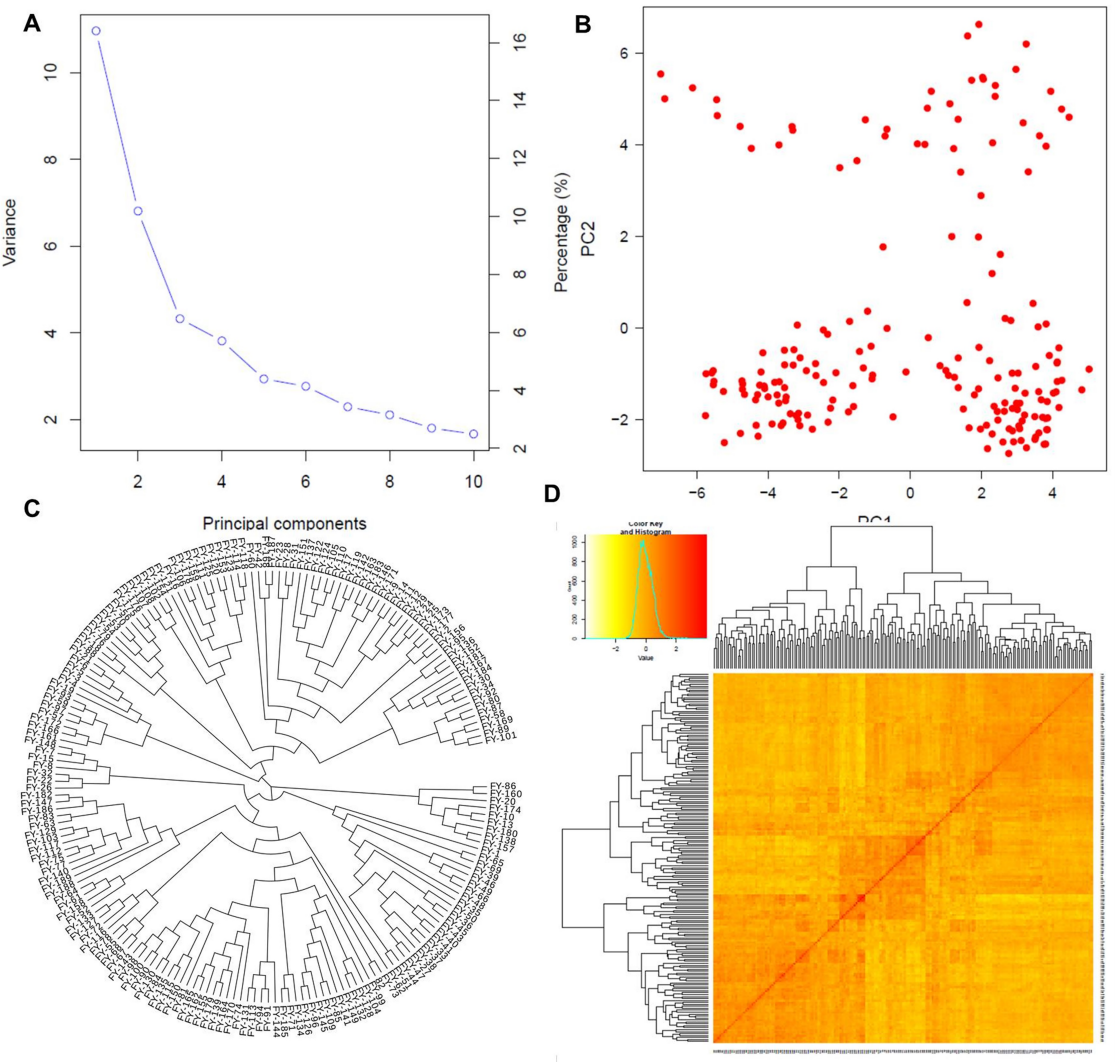
Population structure analysis. Population structure based on principal component analysis (A and B), the phylogenetic relationship among individuals (C), and the kinship matrix among them (D).

The posterior probability of data, the LnP(D) scores for the number of populations (K) increased continuously from 1 to 3 and showed the inflation point at 3 (Table 3 and Fig. 2A), which split up the entire panel into three subgroups (Fig. 3). Therefore, 189 genotypes were divided into three sub-populations. These included a smaller subpopulation of 39 individuals (P1), and a major group of 76 individuals (P2) followed by a subpopulation of 74 genotypes (P3) (Fig. 2B). The subpopulation P3 also includes individuals with genetic admixture from the other two populations which may be due to the breeding activities at various research institutes. On the other hand, most of the genotypes were pure (Figs. 2B and 2C).

**Table 3:** The posterior probability of data, the LnP(D) scores for the number of populations (K) identified in the structure program.

K	Reps	Mean LnP(K)	Stdev LnP(K)	Ln′(K)	|Ln″(K)|	Delta K
1	3	−9,539.8667	0.4726	NA	NA	NA
2	3	−8,356.8000	2.6058	1,183.066667	496.433333	190.513628
3	3	−7,670.167	1.658	686.633	386.300	237.626
4	3	−7,369.8333	1.7039	300.333333	120.733333	70.856344
5	3	−7,190.2333	51.0063	179.600000	29.700000	0.582281
6	3	−6,980.9333	35.9225	209.300000	537.566667	14.964638
7	3	−7,309.2000	788.1079	−328.266667	841.300000	1.067493
8	3	−6,796.1667	108.8041	513.033333	381.700000	3.508141
9	3	−6,664.8333	15.2749	131.333333	845.133333	55.328150
10	3	−7,378.6333	582.5258	−713.800000	NA	NA

#### Principal component analysis

The first two PCs denoted as PC1 and PC2, explained the maximum variation among individuals as shown in Fig. 3A. PC1 divided the population into two distinct groups, while PC2 revealed a group of admixed germplasm (Fig. 3B). These results are consistent with the population genetic structure revealed by kinship analysis using K-matrix in GAPIT (Fig. 3D). Notably, the first two PCs account for the maximum variation in the dataset.

#### Phylogenetic analysis

The phylogenetic analysis divided the entire panel into three clades, similar to the population structure analysis (Fig. 3C). The first clade with nine genotypes represented the ancient and parental genotypes at the root of the phylogenetic tree while the other two bigger clades with maximum population sizes showed mixed genetic information and allelic combinations among the individuals.

### Experiment III: meta-data analysis

#### Marker traits association

**Figure 4:**
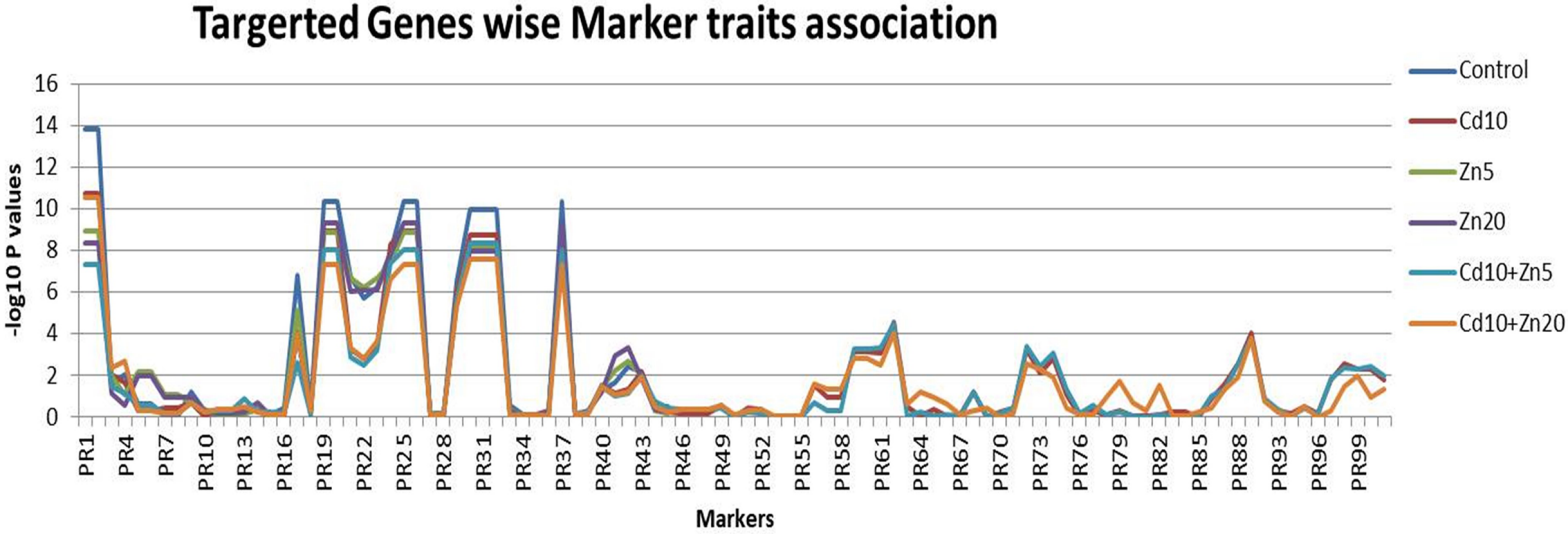
Marker traits association. Scores for 101 genetic markers and the 189 individuals for Zn, Fe and Cd accumulation evaluated under controlled (untreated) conditions and five treatments (Cd_10_, Zn_5_, Zn_20_, Cd_10_+Zn_5_, Cd_10_+Zn_20_).

The statistical regression-based markers traits association was investigated (Fig. 4). The genetic markers were first classified as per their targeted traits such as the nutrient’s accumulations. A total of 18 markers were associated with Zn accumulation in plant tissues. Similarly, 27 and 56 genetic markers were associated with Fe and Cd accumulation, respectively. Out of the 18 markers associated with Zn accumulation, marker 1 and marker 2 were found to be highly associated with this trait, with the highest –log10 *P*-values (ranging from 7.34 to 13.83) across all five treatments (control, Cd_10_, Zn_5_, Zn_20_, Cd_10_+Zn_5_, Cd_10_+Zn_20_). Similarly, among the 27 markers associated with Fe accumulation, the maximum association with the trait was observed for marker 19 and marker 20 (−log10 *P* values range 8.86–10.37) followed by markers 30, 31, and 32 (with –log10 *P* values range 7.6–9.97). Among the evaluated markers for Cd accumulation, two markers 90 and 91 did not show any genetic polymorphism. Therefore, the marker-traits association analysis was performed by using the information from 54 selected markers. Among the studied markers, marker 62 was the most associated (−log10 *P* value 4.00–4.54) followed by marker 89 for Cd accumulation in wheat plants (Data S5). The maximum number of significant associations was found for Fe accumulation in the wheat population. Among the evaluated markers for Fe accumulation, five markers (19, 20, 25, 26, and 37) were the most associated (−log10 *P* value 10.374) followed by markers 30, 31, and 32 for Fe accumulation (Data S5).

#### Germplasm selection

In light of the genotypic and phenotypic results, the top 20 wheat accessions for both Zn and Fe accumulation traits were selected. Among the pool of 40 genotypes with high Zn and Fe accumulation, eight were prominently selected for future breeding and research and were common to both pools (Table 4). The genotype FA-40 exhibited the highest score for Fe accumulation, while FA-35 showed the maximum Zn accumulation.

**Table 4:** List of top eight genotypes, commonly selected from top pools of twenty genotypes for Zn and Fe accumulations which were extracted from a top diverse population of 189 wheat genotypes.

Genotype ID	Iron (mg/kg)	Zn (mg/kg)
FA-40	371.026	46.976
FA-38	186.468	47.117
FA-46	178.111	46.533
FA-41	126.906	46.81
FA-39	120.024	47.27
FA-37	115.389	47.975
FA-36	115.327	49.480
FA-35	99.491	67.504

### Bioavailability of Fe and Zn

**Figure 5:**
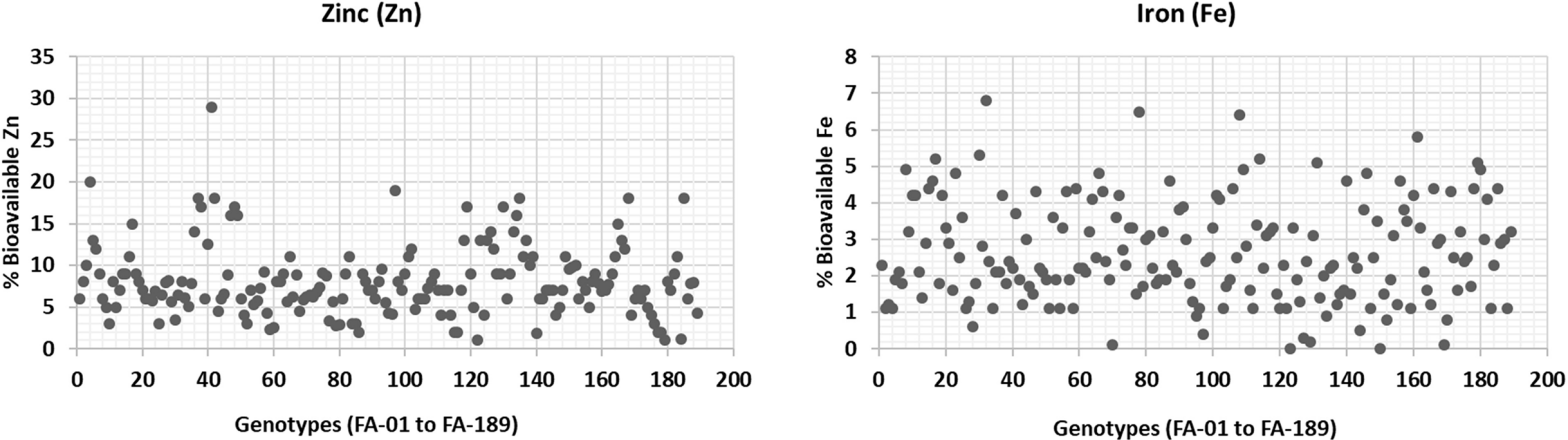
Percent bioavailability of Fe and Zn from wheat grains.

The bioavailability of Zn and Fe was estimated in wheat plants grown under normal conditions (Fig. 5). Zn bioavailability ranged from a minimum of 1% (FA-179, Sehar-2006) to 29% (FA-41). Similarly, Fe bioavailability showed significant variation, ranging from 6.8% (FA-32) to almost 0.01% (FA-150, Satluj-86). Although these findings are consistent with the literature. It is worth noting that the bioavailability of Zn and Fe is significantly influenced by various factors, including antinutrients and environmental conditions (Welch & Graham, 2004; White & Broadley, 2005). Therefore, breeding or bio-fortification programs are generally expected to focus on increasing both the accumulation of micronutrients and their bioavailability (Cakmak, Pfeiffer & McClafferty, 2010).

## Discussion

Biofortified wheat has the potential to overcome malnutrition in targeted areas, as it is a staple food. Significant efforts have been made to explore the genetic diversity in wheat germplasm at both local and international levels. This study aimed to identify germplasm suitable for biofortification, especially by evaluating domestic germplasm using SSR markers associated with the accumulation of Zn, Fe, and Cd in wheat. The collection of wheat germplasm nationwide was also evaluated for Zn, Fe, and Cd accumulation. Molecular markers, such as AFLP, RFLP, RAPD, and SSR are commonly used to investigate the genetic diversity and population structure of various crops including wheat. These markers are specifically used in wheat for phylogenetic investigations, high-resolution mapping, fingerprinting, and marker-assisted selection. Among them, SSRs were selected for this study due to their high polymorphism level and repeatability. Several researchers have used SSR markers to assess the genetic diversity of wheat. In total, 45 SSR primer pairs were used in this study and each pair yielded 101 alleles. Amplified fragments from the primer pairs were analyzed statistically. This technique is commonly used for polyploid species like *B. napus* (Hasan et al., 2008; Xiao et al., 2012), *Arachis* (Huang et al., 2012), and sweet potatoes (Yang et al., 2015).

### Population structure

Cluster analysis is an effective method to evaluate genetic diversity based on the genetic history of genotypes in a well-organized way. In this study, structure analysis was used to cluster the 189 wheat genotypes into three sub-groups, which were consistent with results of PC and kinship analysis, as well as with phylogenetic study. This trend of uniformity established relationships and differentiation between populations indicating gene flow among the collection locations. Genetic diversity can also be boosted by introducing cultivated lines and crossbreeding of genotypes (Camadro, 2012). In the historical breeding program, which was regularly modified and resulted in a narrower genetic base of quality seeds, the three sub-populations clearly identified the ideal parental lines from different collection areas. However, the non-preferred varieties continued to participate in cultivar development, suggesting the presence of certain advantageous alleles that might be investigated by additional research. According to population structure, kinships, and phylogenetic relations among the genotypes, the genotypes at the root of phylogenetic tree may be considered the ancestors of remaining improved cultivars. The genotypes in a common clade may be considered as the improved varieties acquired from the breeding of common ancestors. The degree of genetic association and diversity bestows statistics of diverse genetic history of genotypes. Hence, the information helps in the selection of genetically diverse and well-domesticated plants for future breeding programs.

### The marker-trait association for marker-assisted selection

Our results suggest that the selection of the best suitable breeding lines and their manipulation in breeding programs can effectively improve mineral nutrient content in wheat cultivars. The selection of genotypes can be aided by molecular markers associated with the traits of interest. In this study, initially, we identified SSR markers related to Zn, Fe, and Cd accumulation genes and screened the germplasm, identifying the top associated markers were identified. These markers will be used in further screening the local wheat germplasm before adding it to the breeding program. Additionally, these markers will help to design a strategy for markers-assisted selection during any wheat breeding program, providing a minimum threshold to improve the quality of breeding lines. Examining and considering each component character involved in establishing complex is the best way to study the genetics of complex traits. In future breeding programs, it would be feasible to enhance nutrient absorption in wheat cultivars by introducing the best alleles of component traits linked to the characteristics under investigation into a healthy cultivar.

### Genetic selection

The primary data analysis, including frequency distribution, data skewness, and kurtosis revealed a normal distribution of frequency distribution among genotypes for Zn. The normal distribution indicated maximum diversity and the maximum available alleles regarding the traits, thus showing the population’s best suitability for genome-wide association study. This variation was further confirmed by significantly high variation in analysis of variance (ANOVA) and high coefficients of variation (Data S3). Conversely, the population’s descriptive and frequency distribution for Fe-accumulation revealed a wider range and highly skewed data with very sharp kurtosis, indicating the availability of at least one significant allele dominantly present in the population and associated with Fe accumulation. Genome-wide association, along with other gene mapping tools, can help to reveal the significant allele(s). Although there was a significant variation among genotypes, the within-genotype variation was not significant. Moreover, a higher level of broad sense heritability was observed which allowed us to select the beneficial alleles for the next breeding program. The selected genotypes along with other germplasm, will be further evaluated in field conditions and will proceed further for biofortification in wheat. The present study suggested that the enhancement of mineral nutrients in wheat cultivars can be carried out by selecting suitable breeding lines and manipulating them in breeding programs. The selection of genotypes can be aided by molecular markers significantly associated with the traits of interest.

## Conclusions

Wheat biofortification for Zn and Fe is necessary to enhance the nutritional value of domestic wheat genotypes. A total of 18, 27, and 56 primer pairs were designed to screen the Zn, Fe, and Cd accumulation-related genes. Genotyping of 189 wheat accession revealed a total of 101 alleles across 45 markers. Primers marker 01 and marker 02 were found to be associated with Zn accumulation, while markers represented by numbers 19, 20, 25, 26, 30, 31, 32, and 37 showed significant associations with Fe accumulation-related genes. Markers numbered 62 and 89 were linked to Cd accumulation-related genes. The highly associated markers can be used in a marker-assisted selection of suitable wheat genotypes for breeding biofortified varieties. Moreover, genotypes with better performance are expected to yield better results in field conditions.

## Supplemental Information

10.7717/peerj.15229/supp-1 Supplemental Information 1 Complete list of genotypes.

10.7717/peerj.15229/supp-2 Supplemental Information 2 Fe, Zn and Cd transportation genes.

10.7717/peerj.15229/supp-3 Supplemental Information 3 Statistical description of the Zn and Fe accumulation in the highly diverse population of 189 wheat genotypes. **Indicates the highly significant values (*P* < 0.0001), “ns” indicates non-significant values

10.7717/peerj.15229/supp-4 Supplemental Information 4 Complete list of primer sequences and SSR markers linked to Zn, Fe and Cd.

10.7717/peerj.15229/supp-5 Supplemental Information 5 Markers-traits association revealed by TASSEL and GAPIT analysis for 101 genetic markers and 189 individuals for nutrients accumulation including Zn, Fe, and Cd.

10.7717/peerj.15229/supp-6 Supplemental Information 6 Averaged raw data of Zn and Fe concentration (mg/Kg) obtained from 189 wheat genotypes in three replications.

10.7717/peerj.15229/supp-7 Supplemental Information 7 Agronomic traits data obtained from 189 wheat genotypes.

10.7717/peerj.15229/supp-8 Supplemental Information 8 Genotyping data of 189 genotypes for 101 molecular markers.

## Additional Information and Declarations

### Data Availability

The following information was supplied regarding data availability: The data is available in the Supplemental Files.
