# Supplementary material for: Microsatellite markers-aided dissection of iron, zinc and cadmium accumulation potential in Triticum aestivum
Source: PeerJ. 2023 Apr 17;11:e15229. doi: 10.7717/peerj.15229 (PMC10117381; doi:10.7717/peerj.15229)
Supplement: Supplemental Information 1 [file peerj-11-15229-s001.docx]

Complete list of genotypes.

| **Entry No.** | **Code name** | **Identity of Plant** | **Entry No.** | **Code name** | **Identity of Plant** | **Entry No.** | **Code name** | **Identity of Plant** |
| --- | --- | --- | --- | --- | --- | --- | --- | --- |
| **1** | FA-1 | *AARI-GP1 | **64** | FA-64 | AARI-GP64 | **127** | FA-127 | Pari-73 |
| **2** | FA-2 | AARI-GP2 | **65** | FA-65 | AARI-GP65 | **128** | FA-128 | Punjab-85 |
| **3** | FA-3 | AARI-GP3 | **66** | FA-66 | AARI-GP66 | **129** | FA-129 | AS-2002 |
| **4** | FA-4 | AARI-GP4 | **67** | FA-67 | AARI-GP67 | **130** | FA-130 | FSD-83 |
| **5** | FA-5 | AARI-GP5 | **68** | FA-68 | AARI-GP68 | **131** | FA-131 | Chenab-70 |
| **6** | FA-6 | AARI-GP6 | **69** | FA-69 | ZINCOL-16 | **132** | FA-132 | MH-97 |
| **7** | FA-7 | AARI-GP7 | **70** | FA-70 | AARI-GP68 | **133** | FA-133 | Iqbal-2000 |
| **8** | FA-8 | AARI-GP8 | **71** | FA-71 | AARI-GP69 | **134** | FA-134 | Durum- 97 |
| **9** | FA-9 | AARI-GP9 | **72** | FA-72 | AARI-GP70 | **135** | FA-135 | SH-2002 |
| **10** | FA-10 | AARI-GP10 | **73** | FA-73 | AARI-GP71 | **136** | FA-136 | Yecora-70 |
| **11** | FA-11 | AARI-GP11 | **74** | FA-74 | AARI-GP72 | **137** | FA-137 | UQAB-2000 |
| **12** | FA-12 | AARI-GP12 | **75** | FA-75 | AARI-GP73 | **138** | FA-138 | Ujala-16 |
| **13** | FA-13 | AARI-GP13 | **76** | FA-76 | AARI-GP74 | **139** | FA-139 | Dirk |
| **14** | FA-14 | AARI-GP14 | **77** | FA-77 | AARI-GP75 | **140** | FA-140 | MH-21 |
| **15** | FA-15 | AARI-GP15 | **78** | FA-78 | AARI-GP76 | **141** | FA-141 | Pari-73 |
| **16** | FA-16 | AARI-GP16 | **79** | FA-79 | AARI-GP77 | **142** | FA-142 | Punjab-81 |
| **17** | FA-17 | AARI-GP17 | **80** | FA-80 | AARI-GP78 | **143** | FA-143 | Rothas-90 |
| **18** | FA-18 | AARI-GP18 | **81** | FA-81 | AARI-GP79 | **144** | FA-144 | Pasban-90 |
| **19** | FA-19 | AARI-GP19 | **82** | FA-82 | AARI-GP80 | **145** | FA-145 | AAS-2011 |
| **20** | FA-20 | AARI-GP20 | **83** | FA-83 | AARI-GP81 | **146** | FA-146 | Wadanak-85 |
| **21** | FA-21 | AARI-GP21 | **84** | FA-84 | AARI-GP82 | **147** | FA-147 | FSD-85 |
| **22** | FA-22 | AARI-GP22 | **85** | FA-85 | AARI-GP83 | **148** | FA-148 | Millat-2011 |
| **23** | FA-23 | AARI-GP23 | **86** | FA-86 | AARI-GP84 | **149** | FA-149 | FSD-2008 |
| **24** | FA-24 | AARI-GP24 | **87** | FA-87 | AARI-GP85 | **150** | FA-150 | Kohinoor-83 |
| **25** | FA-25 | AARI-GP25 | **88** | FA-88 | AARI-GP86 | **151** | FA-151 | Seher-2006 |
| **26** | FA-26 | AARI-GP26 | **89** | FA-89 | AARI-GP87 | **152** | FA-152 | Dilkash-21 |
| **27** | FA-27 | AARI-GP27 | **90** | FA-90 | AARI-GP88 | **153** | FA-153 | SA-75 |
| **28** | FA-28 | AARI-GP28 | **91** | FA-91 | AARI-GP89 | **154** | FA-154 | Shafaq-2006 |
| **29** | FA-29 | AARI-GP29 | **92** | FA-92 | AARI-GP90 | **155** | FA-155 | Subhani-21 |
| **30** | FA-30 | AARI-GP30 | **93** | FA-93 | AARI-GP91 | **156** | FA-156 | Chakwal-86 |
| **31** | FA-31 | AARI-GP31 | **94** | FA-94 | AARI-GP92 | **157** | FA-157 | Durum- 21 |
| **32** | FA-32 | AARI-GP32 | **95** | FA-95 | AARI-GP93 | **158** | FA-158 | Galaxy-13 |
| **33** | FA-33 | AARI-GP33 | **96** | FA-96 | AARI-GP94 | **159** | FA-159 | Lasani-2008 |
| **34** | FA-34 | AARI-GP34 | **97** | FA-97 | AARI-GP95 | **160** | FA-160 | Punjab-2011 |
| **35** | FA-35 | AARI-GP35 | **98** | FA-98 | AARI-GP96 | **161** | FA-161 | Punjab-96 |
| **36** | FA-36 | AARI-GP36 | **99** | FA-99 | AARI-GP97 | **162** | FA-162 | AARI-2011 |
| **37** | FA-37 | AARI-GP37 | **100** | FA-100 | AARI-GP98 | **163** | FA-163 | Anaj-17 |
| **38** | FA-38 | AARI-GP38 | **101** | FA-101 | AARI-GP99 | **164** | FA-164 | Inqilab-91 |
| **39** | FA-39 | AARI-GP39 | **102** | FA-102 | AARI-GP100 | **165** | FA-165 | Blue Silver |
| **40** | FA-40 | AARI-GP40 | **103** | FA-103 | AARI-GP101 | **166** | FA-166 | WL-711 |
| **41** | FA-41 | AARI-GP41 | **104** | FA-104 | AARI-GP102 | **167** | FA-167 | Chenab-2000 |
| **42** | FA-42 | AARI-GP42 | **105** | FA-105 | AARI-GP103 | **168** | FA-168 | Akbar-19 |
| **43** | FA-43 | AARI-GP43 | **106** | FA-106 | AARI-GP104 | **169** | FA-169 | Shahkar-95 |
| **44** | FA-44 | AARI-GP44 | **107** | FA-107 | AARI-GP105 | **170** | FA-170 | Punjab- 76 |
| **45** | FA-45 | AARI-GP45 | **108** | FA-108 | AARI-GP106 | **171** | FA-171 | Chilero |
| **46** | FA-46 | AARI-GP46 | **109** | FA-109 | AARI-GP107 | **172** | FA-172 | Satluj-86 |
| **47** | FA-47 | AARI-GP47 | **110** | FA-110 | AARI-GP108 | **173** | FA-173 | Shalimar-88 |
| **48** | FA-48 | AARI-GP48 | **111** | FA-111 | AARI-GP109 | **174** | FA-174 | Watan |
| **49** | FA-49 | AARI-GP49 | **112** | FA-112 | AARI-GP110 | **175** | FA-175 | Cham-06 |
| **50** | FA-50 | AARI-GP50 | **113** | FA-113 | AARI-GP111 | **176** | FA-176 | Ning-8319 |
| **51** | FA-51 | AARI-GP51 | **114** | FA-114 | AARI-GP112 | **177** | FA-177 | Galvez |
| **52** | FA-52 | AARI-GP52 | **115** | FA-115 | AARI-GP113 | **178** | FA-178 | Punjan-96 |
| **53** | FA-53 | AARI-GP53 | **116** | FA-116 | AARI-GP114 | **179** | FA-179 | HP-1744 |
| **54** | FA-54 | AARI-GP54 | **117** | FA-117 | AARI-GP115 | **180** | FA-180 | Saleem-2000 |
| **55** | FA-55 | AARI-GP55 | **118** | FA-118 | AARI-GP116 | **181** | FA-181 | Bhakkar |
| **56** | FA-56 | AARI-GP56 | **119** | FA-119 | AARI-GP117 | **182** | FA-182 | Ufaq-2002 |
| **57** | FA-57 | AARI-GP57 | **120** | FA-120 | AARI-GP118 | **183** | FA-183 | GA-2002 |
| **58** | FA-58 | AARI-GP58 | **121** | FA-121 | Kohistan-97 | **184** | FA-184 | Manthar |
| **59** | FA-59 | AARI-GP59 | **122** | FA-122 | Parwaz-94 | **185** | FA-185 | Marvi-2000 |
| **60** | FA-60 | AARI-GP60 | **123** | FA-123 | MexiPak-65 | **186** | FA-186 | Johar |
| **61** | FA-61 | AARI-GP61 | **124** | FA-124 | Sandal-73 | **187** | FA-187 | Fakhar Bhakar |
| **62** | FA-62 | AARI-GP62 | **125** | FA-125 | Barani-83 | **188** | FA-188 | Fareed-2006 |
| **63** | FA-63 | AARI-GP63 | **126** | FA-126 | Pak-81 | **189** | FA-189 | Miraj-08 |

*AARI-GP represent lines obtained from Ayyub Agriculture Research Institute (AARI).
