# Supplementary material for: Microsatellite markers-aided dissection of iron, zinc and cadmium accumulation potential in Triticum aestivum
Source: PeerJ. 2023 Apr 17;11:e15229. doi: 10.7717/peerj.15229 (PMC10117381; doi:10.7717/peerj.15229)
Supplement: Supplemental Information 2 [file peerj-11-15229-s002.docx]

**Fe, Zn and Cd transportation geness**

| **Sr #** | **Genes name** | **Gene IDs** | **Sr #** | **Genes name** | **Gene IDs** |
| --- | --- | --- | --- | --- | --- |
| **1** | bZIP28 | \| TraesCS7D02G413300.1 \| \| --- \| | **26** | bHMA2-7A-1 | [TraesCS7A02G419500](http://plants.ensembl.org/Triticum_aestivum/Gene/Summary?db=core;g=TraesCS7A02G419500;tl=DZ3DKAl4VbMlB9T6-20615014-1913202358) |
| **2** | bZIP23 | TraesCS4D02G277100.1 | **27** | bHMA2-7B1 | [TraesCS7B02G320100](http://plants.ensembl.org/Triticum_aestivum/Gene/Summary?db=core;g=TraesCS7B02G320100;tl=DZ3DKAl4VbMlB9T6-20615014-1913202319) |
| **3** | bZIP22 | TraesCS4B02G278600.1 | **28** | bHMA2-7D1 | [TraesCS7D02G412400](http://plants.ensembl.org/Triticum_aestivum/Gene/Summary?db=core;g=TraesCS7D02G412400;tl=DZ3DKAl4VbMlB9T6-20615014-1913202342) |
| **4** | bZIP21 | TraesCS4A02G025400.1 | **29** | bHMA3-5A1 | TraesCS5A02G383400 |
| **5** | bZIP20 | TraesCS2D02G506300.1 | **30** | bHMA3-5B1 | [TraesCS5B02G388000](http://plants.ensembl.org/Triticum_aestivum/Gene/Summary?g=TraesCS5B02G388000;r=5B:567018067-567021814;t=TraesCS5B02G388000.1;db=core) |
| **6** | bZIP1-D | TraesCS3D02G532400.1 | **31** | bHMA3-5D1 | [TraesCS5D02G392700](http://plants.ensembl.org/Triticum_aestivum/Gene/Summary?g=TraesCS5D02G392700;r=5D:460941086-460944519;t=TraesCS5D02G392700.1;db=core) |
| **7** | bZIP1-A | TraesCS3A02G527000.1 | **32** | bNRAMP1-7A | TraesCS7A02G327300 |
| **8** | bZIP19 | TraesCS2D02G506200.1 | **33** | bNRAMP1-7B | TraesCS7B02G227900 |
| **9** | bZIP1 | TraesCS2A02G424200.1 | **34** | bNRAMP1-7D | [TraesCS7D02G324000](http://plants.ensembl.org/Triticum_aestivum/Gene/Summary?g=TraesCS7D02G324000;r=7D:413690050-413693308;t=TraesCS7D02G324000.1;db=core) |
| **10** | bNAM-B1 | TraesCS6A02G108300.1 | **35** | bNRAMP2-4A | TraesCS4A02G050500 |
| **11** | bVIT1-2D | TraesCS2D02G326300 | **36** | bNRAMP2-4B | TraesCS4B02G254300 |
| **12** | bVTL4-4D | TraesCS4D02G110600 | **37** | bNRAMP2-4D | [TraesCS4D02G254100](http://plants.ensembl.org/Triticum_aestivum/Gene/Summary?g=TraesCS4D02G254100;r=4D:422959834-422963536;t=TraesCS4D02G254100.1;db=core) |
| **13** | bVTL5-2B_5 | TraesCS2B02G455000 | **38** | bNRAMP3-7A | [TraesCS7A02G464300](http://plants.ensembl.org/Triticum_aestivum/Gene/Summary?g=TraesCS7A02G464300;r=7A:660550059-660555686;t=TraesCS7A02G464300.1;db=core) |
| **14** | bVTL5-2D_3 | TraesCS2D02G588000 | **39** | bNRAMP3-7B | [TraesCS7B02G364800](http://plants.ensembl.org/Triticum_aestivum/Gene/Summary?g=TraesCS7B02G364800;r=7B:627982515-627987790;t=TraesCS7B02G364800.1;db=core) |
| **15** | bVTL2-2A | TraesCS2A02G387100 | **40** | bNRAMP3-7D | [TraesCS7D02G451900](http://plants.ensembl.org/Triticum_aestivum/Gene/Summary?g=TraesCS7D02G451900;r=7D:571715489-571721187;t=TraesCS7D02G451900.1;db=core) |
| **16** | bVTL1-6A | TraesCS6A02G238800 | **41** | bNRAMP4-U1 | [TraesCSU02G077000](http://plants.ensembl.org/Triticum_aestivum/Gene/Summary?g=TraesCSU02G077000;r=Un:69191534-69193788;t=TraesCSU02G077000.1;db=core) |
| **17** | bVTL5-2B_2 | TraesCS2B02G455200 | **42** | bNRAMP5-4B | TraesCS4B02G300600 |
| **18** | bVTL4-4A | TraesCS4A02G209600 | **43** | bNRAMP5-4A | TraesCS4A02G004400 |
| **19** | bVTL5-2B_6 | TraesCS2B02G610400 | **44** | bNRAMP5-4D | TraesCS4D02G299400 |
| **20** | bVTL5-2B_1 | TraesCS2B02G455100 | **45** | LCT1 | AF015523.1 |
| **21** | bVIT2-5D | TraesCS5D02G209900 |  |  |  |
| **22** | bVTL1-6B | TraesCS6B02G285700 |  |  |  |
| **23** | bVTL4-4B | TraesCS4B02G112900 |  |  |  |
| **24** | bVTL2-2D_1 | TraesCS2D02G383900 |  |  |  |
| **25** | bVTL5-2B_4 | TraesCS2B02G455300 |  |  |  |
