# Supplementary material for: Microsatellite markers-aided dissection of iron, zinc and cadmium accumulation potential in Triticum aestivum
Source: PeerJ. 2023 Apr 17;11:e15229. doi: 10.7717/peerj.15229 (PMC10117381; doi:10.7717/peerj.15229)
Supplement: Supplemental Information 3 — **Indicates the highly significant values (P < 0.0001), “ns” indicates non-significant values [file peerj-11-15229-s003.docx]

Statistical description of the Zn and Fe accumulation in the highly diverse population of 189 wheat genotypes.

|  | Iron | Zn |
| --- | --- | --- |
| Minimum | 6.8 | 4.56 |
| Average ± SD | 53.3 ± 50.48 | 33.69 ± 9.48 |
| Maximum | 384.13 | 71.1 |
| Skewness ± SE | 4.665 ± 0.103 | 0.07 ± 0.013 |
| Kurtosis ± SE | 27.74 ± 0.205 | 1.646 ± 0.205 |
| Heritability | 99.98 % | 99.87 % |
| Coefficient of Variation (CV) | 94.71 % | 28.15 % |
| Between-group Variance | 7669.44 ** | 781.11** |
| Within group Variance | 1.504 ns | 0.346 ns |

** indicates the highly significant values (P<0.0001), “ns” indicates non-significant values
