# Supplementary material for: Microsatellite markers-aided dissection of iron, zinc and cadmium accumulation potential in Triticum aestivum
Source: PeerJ. 2023 Apr 17;11:e15229. doi: 10.7717/peerj.15229 (PMC10117381; doi:10.7717/peerj.15229)
Supplement: Supplemental Information 4 [file peerj-11-15229-s004.docx]

**List of primer sequences and SSR markers linked to Zn, Fe and Cd**

| **Gene name** | **PR #** |  | **Primer sequence** | **SSR** | **Tm** | **Len** | **Product size** |
| --- | --- | --- | --- | --- | --- | --- | --- |
|  |  |  |  | **Zn** |  |  |  |
|  | **PR1** | FORWARD | GCTTTCATGAGAAATACAACC | CCACCACCACCACCA | 54.6 | 21 | 152 |
|  |  | REVERSE | AAAAACAGAAAAACTGGCAAC |  | 56.15 | 21 |  |
|  | **PR2** | FORWARD | TGCAGCCAATGAAAAAGCAC | TGCATGCATGCA | 62.2 | 20 | 126 |
| **NAM-B1** |  | REVERSE | TTATAAGGCCCCGCTTTGG |  | 61.1 | 19 |  |
|  | **PR3** | FORWARD | CTCAAAACCAACTGGATCAT | CCACCACCACCA | \| 55.03 \| \| --- \| \|  \| | 20 | 198 |
|  |  | REVERSE | CAACCATCAACAATACCCTAA |  | 55.13 | 21 |  |
| **ZIP28** | **PR4** | FORWARD | ACTGATCAGCAATGTCAACTT | GAGAGAGAGAGAGAGAGAGAGA | 54.85 | 21 | 134 |
|  |  | REVERSE | GCTCAACATTCTGCTTAGAAA |  | 55.02 | 21 |  |
| **ZIP23** | **PR5** | FORWARD | GAGACAGCGAGTTATTGGTT | AGAGAGAGAGAGAGAGAGAGAGAGAGAGAGAGAGAGAGAGAGAGAGAGAGAGAGAG | 54.52 | 20 | 150 |
|  |  | REVERSE | TTCAAACTCAAGTAGTGGATGA |  | 55.04 | 22 |  |
| **ZIP22** | **PR6** | FORWARD | GACGACAGCGATGATGAC | CGCCGCCGCCGC | 55.86 | 18 | 153 |
|  |  | REVERSE | GAATAACGCGCAGAGAGA |  | 55.06 | 18 |  |
|  | **PR7** | FORWARD | ATGCATGTGTCAGGTAGAGAA | AGAGAGAGAGAGAGAGAGAGAGAGAGAGAGAGAGAGAGAGAGAGAGAGAGAG | 55.77 | 21 | 151 |
| **ZIP21** |  | REVERSE | TTTGCACTTTTCTCTGGTAAG |  |  |  |  |
|  | **PR8** | FORWARD | AGTTCATACCCTGATCAATCC | CCTCCTCCTCCT | 55.51 | 21 | 154 |
|  |  | REVERSE | GCAAAGGGAGAGAGGAGA |  | 55.38 | 18 |  |
| **ZIP20** | **PR9** | FORWARD | TGTGCATTTAAGTACAGGAAA | TTTATTTATTTA | 55.26 | 22 | 153 |
|  |  | REVERSE | CGACTTGTACTTCCAGTGAAT |  | 54.46 | 21 |  |
|  | **PR10** | FORWARD | TATGGGAATTGTCACAGTTTT | CATGCATGCATG | 54.67 | 21 | 164 |
|  |  | REVERSE | CAAGTAGTAGCACCTTTGCAT |  | 54.86 | 21 |  |
| **ZIP19** | **PR11** | FORWARD | CACGACTCACTTTTCTTTCAT | ATGCATGCATGC | 54.59 | 21 | 154 |
|  |  | REVERSE | TACTAAGAGCTACTGCGGCTA |  | 55.03 | 21 |  |
|  | **PR12** | FORWARD | AGTAGTCGTACACCCAAACC | ACAACAACAACAACA | 54.14 | 20 | 158 |
|  |  | REVERSE | TCTCCTCTACCCTTCTTCTTC |  | 54.42 | 21 |  |
|  | **PR13** | FORWARD | TACCACCAAGGTAAAAAGACA | ATATATATATATAT | 54.92 | 21 | 137 |
|  |  | REVERSE | GAGGAAAATTCTCAGTCTGTGT |  | 55.01 | 22 |  |
| **ZIP1** | **PR14** | FORWARD | ATGCGTCACGAATACAGTC | CTCCCTCCCTCCCTCC | 54.46 | 19 | 159 |
|  |  | REVERSE | TTAGGCTTTTTCAGGAGTCTT |  | 55.12 | 21 |  |
|  | **PR15** | FORWARD | AACTGAGGCTTAGATGAATCC | ACACACACACACACAC | 55.14 | 21 | 158 |
| **ZIP1-D** |  | REVERSE | CAGTACAGGGCTGACTTTTT |  | 54.62 | 20 |  |
|  | **PR16** | FORWARD | ACCTAAGAAACTCCTCTGCAT | GCTGCTGCTGCT | 54.87 | 21 | 167 |
|  |  | REVERSE | CTGCTGGAATAAGCAGGTA |  | 54.07 | 19 |  |
|  | **PR17** | FORWARD | GGAGGACGTCTAATCTCATTT | GCTGCTGCTGCT | 54.98 | 21 | 126 |
|  |  | REVERSE | CATCTTCGTCCTCCTCATC |  | 55.54 | 19 |  |
| **ZIP1-A** | **PR18** | FORWARD | CACAAGAACACAAGATGGAGT | TACGTACGTACGTACG | 55.2 | 21 | 160 |
|  |  | REVERSE | AGCACGATATATATGCAGAGG |  | 54.67 | 21 |  |
|  |  |  |  | **Fe** |  |  |  |
| **TaVTL5-2B_5** | **PR19** | FORWARD | GCCGAAATGATAACGAAATA | AGCAAGCAAGCA | 55.43 | 20 | 143 |
|  |  | REVERSE | ACAATTGCTAGCGATAATGTG |  | 55.63 | 21 |  |
|  | **PR20** | FORWARD | GGGAGAAACATCATAAGATCC | GAGAGAGAGAGAGAGAGA | 55.15 | 21 | 152 |
|  |  | REVERSE | ACAAAGAGGGAGTAGTTCACC |  | 54.96 | 21 |  |
| **TaVTL5-2B_1** | **PR21** | FORWARD | TACCATGACCGAAATGATAAC | AGCAAGCAAGCA | 55.03 | 21 | 151 |
|  |  | REVERSE | TACAATGGCTAGCGATAATGT |  | 55.15 | 21 |  |
|  | **PR22** | FORWARD | GTACTACCATGACCGAAATGA | AGCAAGCAAGCA | 55.1 | 21 | 180 |
|  |  | REVERSE | GAGTGTACATCATACCGAAGG |  | 54.67 | 21 |  |
|  | **PR23** | FORWARD | TAGTACTACCATGGCCGAAA | AGCAAGCAAGCA | 55.87 | 20 | 156 |
| **TaVTL5-2B_2** |  | REVERSE | TACAATGGCTAGCGATAATGT |  | 55.15 | 21 |  |
|  | **PR24** | FORWARD | TAGTACTACCATGGCCGAAA | AGCAAGCAAGCA | 55.87 | 20 | 181 |
|  |  | REVERSE | GAGTGTACATCATACCGAAGG |  | 54.67 | 21 |  |
| **TaVTL5-2B_4** | **PR25** | FORWARD | AAATTGTCGCTGTTTCACATA | AGCGAGCGAGCG | 55.91 | 21 | 155 |
|  |  | REVERSE | TACAGTTCAAGAACCTTCCAG |  | 54.61 | 21 |  |
|  | **PR26** | FORWARD | CTGGGTGAAGCCGAAAGCTA | TTATTTATTTAT | 62.5 | 20 | 135 |
|  |  | REVERSE | GCCCTAAAGTGCATGTCTGGA |  | 62.7 | 21 |  |
| **TaVIT1-2D** | **PR27** | FORWARD | GCTCAATTCTGTCTGTTGC | GGCGGCGGCGGC | 54.28 | 19 | 165 |
|  |  | REVERSE | GGACACGCCCATGATGAC |  | 60.96 | 18 |  |
|  | **PR28** | FORWARD | GAGATACGTCGAGGAGTTTG | CAGGCAGGCAGGCAGG | 54.94 | 20 | 148 |
|  |  | REVERSE | TCTGAATCTGTTTGCCTTTTA |  | 55.29 | 21 |  |
| **TaVTL4-4D** | **PR29** | FORWARD | TCCATTAGGAGTAGAGATGACC | TGTATGTATGTA | 55 | 22 |  |
|  |  | REVERSE | TATATAGCGACGAGGCAGTAG |  | 55.08 | 21 |  |
| **TaVTL5-2D_3** | **PR30** | FORWARD | TGGAATGGATGGGTTAATAAT | GAGAGAGAGAGAGA | 55.87 | 21 | 158 |
|  |  | REVERSE | GTGTGGCTAGTGCGTACAT |  | 54.6 | 19 |  |
| **TaVTL2-2A** | **PR31** | FORWARD | TCGACTCGACAATTTCTCTAA | TTTGTTTGTTTG | 55.28 | 21 | 147 |
|  |  | REVERSE | TCACTTGTGTATTCCCCTAAA |  | 54.84 | 21 |  |
| **TaVTL1-6A** | **PR32** | FORWARD | GAAGGGAGGAGTAGCAGTAGA | GGCGGCGGCGGCGGC | 55.38 | 21 | 116 |
|  |  | REVERSE | GCCTTGAGGTGGAACTGC |  | 59.35 | 18 |  |
| **TaVTL4-4A** | **PR33** | FORWARD | GCTTCCAATGCCTATATTTCT | GCGCGCGCGCGCGC | 55.27 | 21 | 205 |
|  |  | REVERSE | TTTTAGAGCATCTTCAACAGG |  | 54.88 | 21 |  |
|  | **PR34** | FORWARD | GCTTCCAATGCCTATATTTC | GCGCGCGCGCGCGC | 54.12 | 20 | 203 |
|  |  | REVERSE | TTAGAGCATCTTCAACAGGTG |  | 55.64 | 21 |  |
| **TaVTL5-2B_6** | **PR35** | FORWARD | GGATGGGTTAATAATCTAGGC | GAGAGAGAGAGAGAGA | 54.53 | 21 | 157 |
|  |  | REVERSE | ACTGTGTGGCTAGTGCGTA |  | 55.33 | 19 |  |
|  | **PR36** | FORWARD | GATCGTCGTCTCCTCCAT | TACGTACGTACG | 55.84 | 18 | 177 |
|  |  | REVERSE | GCAAAGACTAGCAAATAGCAG |  | 54.83 | 21 |  |
| **TaVTL2-2D_1** | **PR37** | FORWARD | CTCCTTCTTGAGTAGCTCGAT | CGTCGTCGTCGT | 55.54 | 21 | 189 |
|  |  | REVERSE | GCGGGCCATGTAGTTGAC |  | 60.08 | 18 |  |
|  | **PR38** | FORWARD | GGATCCATGGACCATCAC | CGTCGTCGTCGT | 56.78 | 18 | 274 |
|  |  | REVERSE | ACACGAGCATCGTCTTGC |  | 58.45 | 18 |  |
| **TaVIT2-5D** | **PR39** | FORWARD | CAGGTCACTCTCTCACTTGAC | TCTAATCTAATCTAA | 54.87 | 21 | 149 |
|  |  | REVERSE | CCCGTACTCAGAGAGTATGTC |  | 53.94 | 21 |  |
|  | **PR40** | FORWARD | CTGGATATTTGCATGTGGTT | TGTGTGTGTGTG | 55.96 | 20 | 145 |
|  |  | REVERSE | GAACTTCAGCAGAACAGGTC |  | 54.95 | 20 |  |
| **TaVTL1-6B** | **PR41** | FORWARD | GTGGTGGACGTGTACCTG | GGAGGAGGAGGA | 55.51 | 18 | 147 |
|  |  | REVERSE | CTTGTACGCCCTGTTGAC |  | 55 | 18 |  |
|  | **PR42** | FORWARD | GGGGAGAACAAACTTTACCTA | CCTCCTCCTCCT | 55.09 | 21 | 162 |
|  |  | REVERSE | CTGTAGAGAGCAAAACTCCAT |  | 53.86 | 21 |  |
|  | **PR43** | FORWARD | GTGGTTGGGTAGCTCGTG | GGCGGCGGCGGC | 57.58 | 18 | 165 |
|  |  | REVERSE | AGAGAGACGCCTTTTTCTATC |  | 54.63 | 21 |  |
| **TaVTL4-4B** | **PR44** | FORWARD | AGCTGCACGACTGCAATG | GGCGGCGGCGGC | 59.71 | 18 | 110 |
|  |  | REVERSE | AGAGAGACGCCTTTTTCTATCC |  | 58.19 | 22 |  |
|  | **PR45** | FORWARD | GGGGAAGAGTGAAGAAGAGT | GGAGGGAGGGAG | 54.98 | 20 | 144 |
|  |  | REVERSE | GAGTGAAACGGACAGCAC |  | 54.92 | 18 |  |
|  |  |  |  | **Cd** |  |  |  |
| **HMA2-7A1** | **PR46** | FORWARD | AAATTCTAGCCCATTTAACCA | TCCTCCTCCTCC | 55.59 | 21 | 155 |
|  |  | REVERSE | TGACTTCAGTTTGGATGGTAT |  | 54.64 | 21 |  |
|  | **PR47** | FORWARD | GGACTCCTGGGTGAACTC | CCGCCCCGCCCCGCCCCGCC | 55.22 | 18 | 232 |
|  |  | REVERSE | AGACGAGGAGGAGGATGAT |  | 56.1 | 19 |  |
|  | **PR48** | FORWARD | TCCAGTTGAAACTAATATGCAG | TCATCATCATCATCA | 54.79 | 22 | 120 |
|  |  | REVERSE | AGCTATGAAAACTGTTGTTGG |  | 54.66 | 21 |  |
| **HMA2-7B1** | **PR49** | FORWARD | GGTCCAAGTTTCTATTCACAGT | CACACACACACACA | 55 | 22 | 150 |
|  |  | REVERSE | AACTGCATGAATCTAACAGGA |  | 54.97 | 21 |  |
|  | **PR50** | FORWARD | TTCCTGTTAGATTCATGCAGT | CACACACACACACACACACACACA | 54.97 | 21 | 146 |
|  |  | REVERSE | TCTTTTAGAAAATGGATGAGC |  | 53.91 | 21 |  |
|  | **PR51** | FORWARD | GTTTTGCAAACGAGAGAAGA | ACACACACACAC | 55.75 | 20 | 165 |
|  |  | REVERSE | CCTCTCTTCTTGGGAGTTTT |  | 55.17 | 20 |  |
|  | **PR52** | FORWARD | GCTTGCGTTTACATGAGG | CCTTCCCTTCCCTTCCCTTCCCTTC | 55.29 | 18 | 160 |
|  |  | REVERSE | GAGAGGCGTGATGAACTG |  | 55.03 | 18 |  |
| **HMA2-7D1** | **PR53** | FORWARD | GTAATAGCCCGTGGATGTAG | CTCCTCCTCCTC | 54.81 | 20 | 166 |
|  |  | REVERSE | ATTAACTGGCAAACCCACCTA |  | 58.5 | 21 |  |
|  | **PR54** | FORWARD | CACTCTTCTTGGCTCTCTTG | CGGCGGCGGCGG | 55.33 | 20 | 172 |
|  |  | REVERSE | TCGAAGTAGCTCTTCTCCAG |  | 54.99 | 20 |  |
|  | **PR55** | FORWARD | ATCGGTAAGCAAGCACAC | TCCTCCTCCTCCTCC | 54.64 | 18 | 120 |
|  |  | REVERSE | AGGAACAAGACAAGTTGATGA |  | 54.83 | 21 |  |
| **HMA3-5A1** | **PR56** | FORWARD | GGTGATGGTTGTTAAATTTGT | TTCTTCTTCTTC | 54.11 | 21 | 143 |
|  |  | REVERSE | GAATGTCATGTTTCGAGCTAT |  | 54.47 | 21 |  |
|  | **PR57** | FORWARD | TAGGTTTAAGTGGACCGAGAC | ATAATAATAATAATA | 55.98 | 21 | 151 |
|  |  | REVERSE | AGTCTTGTGAAGTCGCAGTTA |  | 55.29 | 21 |  |
|  | **PR58** | FORWARD | ATAAGACAGCCTTGTCACAGA | GTTTGTTTGTTT | 55.04 | 21 | 174 |
|  |  | REVERSE | CAATAATGCTCGCTCCAC |  | 55.2 | 18 |  |
| **HMA3-5B1** | **PR59** | FORWARD | AGAGGACAGGGCAGAGTG | GAGTGAGAGTGAGAGTGA | 56.22 | 18 | 182 |
|  |  | REVERSE | AGTTAACCCGATTTCTGACA |  | 54.79 | 20 |  |
|  | **PR60** | FORWARD | AATACCATCCTGCATCAAGTA | ACAGACAGACAG | 54.78 | 21 | 161 |
|  |  | REVERSE | AGATGCAGAAACATAGATGGA |  | 54.88 | 21 |  |
|  | **PR61** | FORWARD | GAAAAAGGAGAAAAATCAAGC | AGAAGAAGAAGA | 55.02 | 21 | 102 |
|  |  | REVERSE | TGCAGGTCCTAGTACAGGTAA |  | 55.1 | 21 |  |
|  | **PR62** | FORWARD | GTTGTTTGCTTATTTTGATGC | AAATAAATAAAT | 55.15 | 21 | 152 |
|  |  | REVERSE | CCGTTAGTGAAAGAAAACAAA |  | 54.82 | 21 |  |
| **HMA3-5D1** | **PR63** | FORWARD | ATGATGACATGTTGGTTGTTT | AAGAAAGAAAGA | 55.25 | 21 | 155 |
|  |  | REVERSE | AGTTTTCCACTTCTCCTCATC |  | 55.01 | 21 |  |
|  | **PR64** | FORWARD | TAGTGGAGGGGCTGTAAGATA | GTTTGTTTGTTT | 56.5 | 21 | 160 |
|  |  | REVERSE | GAGCACCGTTGATTAAATTAT |  | 53.7 | 21 |  |
|  | **PR65** | FORWARD | AGCTTTGCTCCTACTGTTGAT | ATGGATGGATGG | 55.88 | 21 | 160 |
|  |  | REVERSE | ATAGTGTGATCGATGTTGCTT |  | 54.83 | 21 |  |
| **TaNRAMP1-7A** | **PR66** | FORWARD | TGTATAATGGCCTTGGTTATC | CAGCAGCAGCAG | 54.35 | 21 | 152 |
|  |  | REVERSE | GTAGTGCATGTGCTTCCTTAC |  | 55.08 | 21 |  |
|  | **PR67** | FORWARD | CTCTCAATGCCTTCTCTTTTT | CGGCGGCGGCGG | 55.51 | 21 | 175 |
|  |  | REVERSE | GTTAGGGAACAAAACCTATCC |  | 54.48 | 21 |  |
|  | **PR68** | FORWARD | GTTTTAAACCATTTTCTGACG | CGCTCGCTCGCT | 54.21 | 21 | 175 |
|  |  | REVERSE | GAAAGGAGGAGTGAGAGAGAG |  | 54.87 | 21 |  |
|  | **PR69** | FORWARD | TGAGCCAGATCACATAAAGTT | GAAGAAGAAGAAGAA | 54.97 | 21 | 164 |
|  |  | REVERSE | TAGGGCATATTTCCAACATC |  | 55.1 | 20 |  |
| **TaNRAMP1-7B** | **PR70** | FORWARD | CTCTCATCCCTCTTCTCAAGT | CAGCAGCAGCAG | 55.17 | 21 | 166 |
|  |  | REVERSE | TAGGTACATGGATGGATTCAG |  | 55.03 | 21 |  |
|  | **PR71** | FORWARD | ACTCAACACATGGAGTGGTAG | CTCTCTCTCTCT | 55.07 | 21 | 152 |
|  |  | REVERSE | CCGCTTGTAACTGTTAGATTT |  | 54 | 21 |  |
| **TaNRAMP1-7D** | **PR72** | FORWARD | ATACTGTCATTTGAGCTACCG | CAGCAGCAGCAG | 54.61 | 21 | 159 |
|  |  | REVERSE | CTTCCACAGCCAAAAATATAA |  | 54.72 | 21 |  |
|  | **PR73** | FORWARD | GGTTTTTCTGTTTTCTTTCG | TTTTCTTTTCTTTTC | 54.28 | 20 | 146 |
|  |  | REVERSE | TGCGAATTAATAGATTTTCG |  | 53.41 | 20 |  |
|  | **PR74** | FORWARD | GTGGGAGATGGAGAGGAG | CGGCGGCGGCGG | 55.33 | 18 | 247 |
|  |  | REVERSE | CCTAACCCTAGCAAAATCG |  | 55.09 | 19 |  |
| **TaNRAMP2-4A** | **PR75** | FORWARD | AAGATAGAGCAGGTTTTCACC | ACCACCACCACC | 55.22 | 21 | 186 |
|  |  | REVERSE | GTTAGGTGGTGTCGAGGTC |  | 55.38 | 19 |  |
|  | **PR76** | FORWARD | GTTTCACGAAGGAGTCAGAG | GGCGGGGGCGGGGGCGGG | 54.97 | 20 | 219 |
|  |  | REVERSE | AGATGGAGATGGAGACTTTGT |  | 55.29 | 21 |  |
|  | **PR77** | FORWARD | ACCGGAACCACTGCCACT | CACCACCACCAC | 61.58 | 18 | 145 |
|  |  | REVERSE | GTGGCCTCCTCTGTCGTG |  | 60.41 | 18 |  |
| **TaNRAMP2-4B** | **PR78** | FORWARD | CCCTCTTCTCTTCCAAATAAC | GAAGAAGAAGAA | 54.74 | 21 | 149 |
|  |  | REVERSE | GTTTGTTCCTCTTTTTCTTGG |  | 55.67 | 21 |  |
|  | **PR79** | FORWARD | GTTCAAAAGGAGGAGACAGAT | TTGATTGATTGA | 55.01 | 21 | 137 |
|  |  | REVERSE | TTGTTGGAACGAATTTATAGC |  | 54.71 | 21 |  |
|  | **PR80** | FORWARD | CACGAAGGAGTTAGAGGAACT | CGCCTCCGCCTCCGCCTC | 55.3 | 21 | 215 |
|  |  | REVERSE | GAGATGGAGATGGAGACCTT |  | 55.62 | 20 |  |
| **TaNRAMP2-4D** | **PR81** | FORWARD | AAGGTCTCCATCTCCATCTC | CGCGCGCGCGCG | 55.62 | 20 | 131 |
|  |  | REVERSE | CCGATGCACATGAGGAAG |  | 58.67 | 18 |  |
|  | **PR82** | FORWARD | CCCTCTTCTTCTCCAAATAAC | CGCCGCCGCCGC | 54.74 | 21 | 158 |
|  |  | REVERSE | TCATTTGCTCCTCTTTTTCTT |  | 55.9 | 21 |  |
|  | **PR83** | FORWARD | CGAACTCAAACTGCATAAGAT | GTTGGTTGGTTG | 54.74 | 21 | 142 |
|  |  | REVERSE | ACCTACCAGAGAAAGGAACAC |  | 54.96 | 21 |  |
|  | **PR84** | FORWARD | GTTCTTCCCCTTGTACTGTTC | CCACCACCACCA | 55.38 | 21 | 140 |
|  |  | REVERSE | GATCTCAAGAACGAACTCACA |  | 55.42 | 21 |  |
| **TaNRAMP3-7A** | **PR85** | FORWARD | GAAAGAGGGAGATAGACGAAA | GAGAGAGAGAGAGAGAGAGA | 55.27 | 21 | 167 |
|  |  | REVERSE | AGAAAGGCGAAACCTAAGTC |  | 55.36 | 20 |  |
| **TaNRAMP3-7B** | **PR86** | FORWARD | GGAGAAGACGAGGTAAGAAGA | CGCCGCCGCCGCCGCCGCCGCCGC | 55.34 | 21 | 131 |
|  |  | REVERSE | GAGACCGGAGAGAGAGGA |  | 55.18 | 18 |  |
|  | **PR87** | FORWARD | ACGCAACTGATAAATACCAAA | TTATTTATTTAT | 55.04 | 21 | 167 |
|  |  | REVERSE | TTGCACGTCAATACTTAATCA |  | 54.5 | 21 |  |
| **TaNRAMP3-7D** | **PR88** | FORWARD | TTATTGTTATTTGGAGGGTAGG | TTGTTTGTTTGTTTGT | 55.1 | 22 | 153 |
|  |  | REVERSE | TAAACTTTAGGGCCTTTATCC |  | 54.54 | 21 |  |
|  | **PR89** | FORWARD | AACCATGAGTCGCTTTTCTA | CGAGAGCGAGAGCGAGAG | 55.12 | 20 | 153 |
|  |  | REVERSE | AAGGTCACCGACCTCTCT |  | 54.48 | 18 |  |
| **TaNRAMP4-U1** | **PR90** | FORWARD | CCCTCTAGCAGATCTCGTC | CGCCGCCGCCGC | 54.99 | 19 | 224 |
|  |  | REVERSE | GTAGGAACCTTCTTGTGCTTC |  | 55.69 | 21 |  |
|  | **PR91** | FORWARD | CCCTCTAGCAGATCTCGTC | CGCCGCCGCCGC | 54.99 | 19 | 224 |
|  |  | REVERSE | GTAGGAACCTTCTTGTGCTTC |  | 55.69 | 21 |  |
| **TaNRAMP5-4B** | **PR92** | FORWARD | ATGATACTGTCGTTTGAGCTG | CAGCAGCAGCAGCAG | 55.5 | 21 | 151 |
|  |  | REVERSE | GAGTACGTGTCCAAAAACAAG |  | 54.97 | 21 |  |
|  | **PR93** | FORWARD | GAGGACCTGGCAGACATAC | GCTAGCTAGCTA | 55.47 | 19 | 160 |
|  |  | REVERSE | CGCACCGTAAAACATATACTC |  | 55.08 | 21 |  |
|  | **PR94** | FORWARD | ATGCACTAGCAAATTAAGCAT | TTCATTCATTCA | 54.49 | 21 | 149 |
|  |  | REVERSE | CAGCTCAAACGACAGTATCAT |  | 55.5 | 21 |  |
| **TaNRAMP5-4A** | **PR95** | FORWARD | CTCCCTCACTGTCAATATCAG | ACACACACACACACACAC | 54.79 | 21 | 148 |
|  |  | REVERSE | TCGTGCGAAATATAGTAGTGG |  | 55.63 | 21 |  |
|  | **PR96** | FORWARD | GACTAGAGGGGGAATAGATGA | CAACAACAACAA | 54.91 | 21 | 155 |
|  |  | REVERSE | ATTGTGTTCGTATCTTGTGCT |  | 54.91 | 21 |  |
| **TaNRAMP5-4D** | **PR97** | FORWARD | ATTAGGGAGAACGAAAATGAC | ACACACACACAC | 55 | 21 | 152 |
|  |  | REVERSE | CTGGCTGCTAGTAAGTCGTAG |  | 54.78 | 21 |  |
|  | **PR98** | FORWARD | CGGTTAACTTGTTTGTTTACG | CTCCTCCTCCTC | 55.21 | 21 | 167 |
|  |  | REVERSE | CAGCATGGAGATGAGAAACT |  | 55.36 | 20 |  |
| **TaLCT1** | **PR99** | FORWARD | ACTCATGGATCAGGACAAAG | GGTTGGTTGGTT | 55.01 | 20 | 147 |
|  |  | REVERSE | TCACATCATTAGACTGCAAGTT |  | 55.07 | 22 |  |
|  | **PR100** | FORWARD | AGTATCAATGAAATCCAGCAA | ACATACATACAT | 54.91 | 21 | 156 |
|  |  | REVERSE | ATAATACAAGGCAACGTGTGT |  | 54.81 | 21 |  |
|  | **PR101** | FORWARD | CTTGTTGGTGAGGAACATCT | GCTGAGCTGAGCTGA | 55.1 | 20 | 139 |
|  |  | REVERSE | CAAAATTATCCCTCCTCTCC |  | 55.31 | 20 |  |
