# Supplementary material for: Microsatellite markers-aided dissection of iron, zinc and cadmium accumulation potential in Triticum aestivum
Source: PeerJ. 2023 Apr 17;11:e15229. doi: 10.7717/peerj.15229 (PMC10117381; doi:10.7717/peerj.15229)
Supplement: Supplemental Information 5 [file peerj-11-15229-s005.docx]

**Supplementary data 5:** Markers-traits association revealed by TASSEL and GAPIT analysis for 101 genetic markers and 189 individuals for nutrients accumulation including Zn, Fe, and Cd.

| Treatments | | | | Control | | | Cd10 | | | Zn5 | |  | Zn20 | |  | Cd10+ | | Zn5 | Cd10+Zn20 | | |
| --- | --- | --- | --- | --- | --- | --- | --- | --- | --- | --- | --- | --- | --- | --- | --- | --- | --- | --- | --- | --- | --- |
| Markers associated to Zn accumulation | | | | | | | | | | | |  | | |  | | |  | | | |
|  | marker | 1 | |  | 13.825 |  |  | 10.776 |  |  | 8.981 |  |  | 8.385 |  |  | 7.338 |  |  | 10.562 |  |
|  | marker | 2 | |  | 13.825 |  |  | 10.776 |  |  | 8.981 |  |  | 8.385 |  |  | 7.338 |  |  | 10.562 |  |
| marker 3 | | | | 1.629 | | | 2.024 | | | 1.984 | |  | 1.159 | |  | 1.473 | |  | 2.388 | | |
| marker 4 | | | | 2.055 | | | 1.677 | | | 1.113 | |  | 0.590 | |  | 1.179 | |  | 2.710 | | |
| marker 5 | | | | 0.636 | | | 0.336 | | | 2.175 | |  | 2.001 | |  | 0.562 | |  | 0.283 | | |
| marker 6 | | | | 0.636 | | | 0.336 | | | 2.175 | |  | 2.001 | |  | 0.562 | |  | 0.283 | | |
| marker 7 | | | | 0.052 | | | 0.460 | | | 1.117 | |  | 0.927 | |  | 0.177 | |  | 0.210 | | |
| marker 8 | | | | 0.052 | | | 0.460 | | | 1.117 | |  | 0.927 | |  | 0.177 | |  | 0.210 | | |
| marker 9 | | | | 1.184 | | | 0.709 | | | 0.674 | |  | 0.927 | |  | 0.675 | |  | 0.672 | | |
| marker 10 | | | | 0.331 | | | 0.076 | | | 0.365 | |  | 0.309 | |  | 0.258 | |  | 0.242 | | |
| marker 11 | | | | 0.149 | | | 0.400 | | | 0.102 | |  | 0.196 | |  | 0.336 | |  | 0.392 | | |
| marker 12 | | | | 0.149 | | | 0.400 | | | 0.102 | |  | 0.196 | |  | 0.336 | |  | 0.392 | | |
| marker 13 | | | | 0.171 | | | 0.465 | | | 0.192 | |  | 0.226 | |  | 0.916 | |  | 0.484 | | |
| marker 14 | | | | 0.378 | | | 0.311 | | | 0.375 | |  | 0.707 | |  | 0.244 | |  | 0.260 | | |
| marker 15 | | | | 0.102 | | | 0.054 | | | 0.069 | |  | 0.058 | |  | 0.227 | |  | 0.091 | | |
| marker 16 | | | | 0.462 | | | 0.064 | | | 0.252 | |  | 0.201 | |  | 0.164 | |  | 0.098 | | |
| marker 17 | | | | 6.854 | | | 4.108 | | | 5.134 | |  | 3.923 | |  | 2.625 | |  | 3.995 | | |
| marker 18 | | | | 0.401 | | | 0.090 | | | 0.279 | |  | 0.364 | |  | 0.145 | |  | 0.345 | | |
| Markers associated to Fe accumulation | | | | | | | | | | | |  | | |  | | |  | | | |
|  | Marker19 | |  |  | 10.374 |  |  | 8.980 |  |  | 8.859 |  |  | 9.349 |  |  | 8.017 |  |  | 7.337 |  |
|  | Marker20 | |  |  | 10.374 |  |  | 8.980 |  |  | 8.859 |  |  | 9.349 |  |  | 8.017 |  |  | 7.337 |  |
| Marker 21 | | | | 6.767 | | | 3.295 | | | 6.675 | |  | 6.070 | |  | 2.899 | |  | 3.366 | | |
| Marker 22 | | | | 5.731 | | | 2.850 | | | 6.258 | |  | 6.133 | |  | 2.481 | |  | 2.825 | | |
| Marker 23 | | | | 6.264 | | | 3.496 | | | 6.720 | |  | 6.117 | |  | 3.187 | |  | 3.645 | | |
| Marker 24 | | | | 7.894 | | | 8.281 | | | 7.400 | |  | 7.768 | |  | 7.392 | |  | 6.602 | | |
|  | Marker25 | |  |  | 10.374 |  |  | 8.980 |  |  | 8.859 |  |  | 9.349 |  |  | 8.017 |  |  | 7.337 |  |
|  | Marker 26 | |  |  | 10.374 |  |  | 8.980 |  |  | 8.859 |  |  | 9.349 |  |  | 8.017 |  |  | 7.337 |  |
| marker 27 | | | | 0.086 | | | 0.163 | | | 0.107 | |  | 0.083 | |  | 0.158 | |  | 0.126 | | |
| Marker 28 | | | | 0.086 | | | 0.163 | | | 0.107 | |  | 0.083 | |  | 0.158 | |  | 0.126 | | |
| Marker 29 | | | | 6.593 | | | 5.937 | | | 5.663 | |  | 5.503 | |  | 5.528 | |  | 5.373 | | |
|  | Marker 30 | |  |  | 9.971 |  |  | 8.760 |  |  | 8.257 |  |  | 7.966 |  |  | 8.399 |  |  | 7.603 |  |
|  | Marker 31 | |  |  | 9.971 |  |  | 8.760 |  |  | 8.257 |  |  | 7.966 |  |  | 8.399 |  |  | 7.603 |  |
|  | Marker 32 | |  |  | 9.971 |  |  | 8.760 |  |  | 8.257 |  |  | 7.966 |  |  | 8.399 |  |  | 7.603 |  |
| Marker 33 | | | | 0.594 | | | 0.131 | | | 0.360 | |  | 0.303 | |  | 0.114 | |  | 0.065 | | |
| Marker 34 | | | | 0.117 | | | 0.104 | | | 0.030 | |  | 0.077 | |  | 0.090 | |  | 0.103 | | |
| Marker 35 | | | | 0.118 | | | 0.038 | | | 0.046 | |  | 0.124 | |  | 0.062 | |  | 0.128 | | |
| Marker 36 | | | | 0.164 | | | 0.230 | | | 0.177 | |  | 0.296 | |  | 0.108 | |  | 0.087 | | |
|  | Marker 37 | |  |  | 10.374 |  |  | 8.980 |  |  | 8.859 |  |  | 9.349 |  |  | 8.017 |  |  | 7.337 |  |
| Marker 38 | | | | 0.086 | | | 0.163 | | | 0.107 | |  | 0.083 | |  | 0.158 | |  | 0.126 | | |
| Marker 39 | | | | 0.290 | | | 0.264 | | | 0.223 | |  | 0.296 | |  | 0.228 | |  | 0.207 | | |
| Marker 40 | | | | 1.276 | | | 1.556 | | | 1.413 | |  | 1.223 | |  | 1.488 | |  | 1.481 | | |
| Marker 41 | | | | 1.673 | | | 1.141 | | | 2.239 | |  | 2.987 | |  | 1.023 | |  | 1.078 | | |
| Marker 42 | | | | 2.416 | | | 1.368 | | | 2.691 | |  | 3.375 | |  | 1.158 | |  | 1.194 | | |

| Marker 43 | | | 2.162 | 2.211 | | | 1.969 | 1.869 | 2.012 | |  | 1.910 | | |
| --- | --- | --- | --- | --- | --- | --- | --- | --- | --- | --- | --- | --- | --- | --- |
| Marker 44 | | | 0.515 | 0.632 | | | 0.338 | 0.407 | 0.763 | |  | 0.530 | | |
| Marker 45 | | | 0.508 | 0.277 | | | 0.215 | 0.218 | 0.443 | |  | 0.272 | | |
| Markers associated to Cd accumulation | | | | | | | | | | |  | | | |
| Marker 46 | | |  | 0.199 | | |  |  | 0.403 | |  | 0.361 | | |
| Marker 47 | | |  | 0.199 | | |  |  | 0.403 | |  | 0.361 | | |
| Marker 48 | | |  | 0.199 | | |  |  | 0.403 | |  | 0.361 | | |
| Marker 49 | | |  | 0.557 | | |  |  | 0.436 | |  | 0.573 | | |
| Marker 50 | | |  | 0.020 | | |  |  | 0.004 | |  | 0.121 | | |
| Marker 51 | | |  | 0.469 | | |  |  | 0.277 | |  | 0.331 | | |
| Marker 52 | | |  | 0.380 | | |  |  | 0.182 | |  | 0.351 | | |
| Marker 53 | | |  | 0.001 | | |  |  | 0.014 | |  | 0.075 | | |
| Marker 54 | | |  | 0.001 | | |  |  | 0.014 | |  | 0.075 | | |
| Marker 55 | | |  | 0.001 | | |  |  | 0.014 | |  | 0.075 | | |
| Marker 56 | | |  | 1.539 | | |  |  | 0.710 | |  | 1.605 | | |
| Marker 57 | | |  | 0.961 | | |  |  | 0.289 | |  | 1.326 | | |
| Marker 58 | | |  | 0.961 | | |  |  | 0.289 | |  | 1.326 | | |
| Marker 59 | | |  | 3.126 | | |  |  | 3.296 | |  | 2.840 | | |
| Marker 60 | | |  | 3.126 | | |  |  | 3.296 | |  | 2.840 | | |
| Marker 61 | | |  | 3.112 | | |  |  | 3.345 | |  | 2.512 | | |
|  | Marker 62 |  |  |  | 4.537 |  |  |  |  | 4.531 |  |  | 4.066 |  |
| Marker 63 | | |  | 0.483 | | |  |  | 0.082 | |  | 0.635 | | |
| Marker 64 | | |  | 0.050 | | |  |  | 0.223 | |  | 1.188 | | |
| Marker 65 | | |  | 0.400 | | |  |  | 0.087 | |  | 0.974 | | |
| Marker 66 | | |  | 0.052 | | |  |  | 0.097 | |  | 0.627 | | |
| Marker 67 | | |  | 0.120 | | |  |  | 0.026 | |  | 0.152 | | |
| Marker 68 | | |  | 1.210 | | |  |  | 1.188 | |  | 0.284 | | |
| Marker 69 | | |  | 0.012 | | |  |  | 0.046 | |  | 0.431 | | |
| Marker 70 | | |  | 0.240 | | |  |  | 0.211 | |  | 0.017 | | |
| Marker 71 | | |  | 0.471 | | |  |  | 0.416 | |  | 0.163 | | |
| Marker 72 | | |  | 3.264 | | |  |  | 3.397 | |  | 2.555 | | |
| Marker 73 | | |  | 2.144 | | |  |  | 2.431 | |  | 2.338 | | |
| Marker 74 | | |  | 2.848 | | |  |  | 3.077 | |  | 1.937 | | |
| Marker 75 | | |  | 1.069 | | |  |  | 1.367 | |  | 0.470 | | |
| Marker 76 | | |  | 0.054 | | |  |  | 0.185 | |  | 0.097 | | |
| Marker 77 | | |  | 0.287 | | |  |  | 0.551 | |  | 0.143 | | |
| Marker 78 | | |  | 0.110 | | |  |  | 0.126 | |  | 0.953 | | |
| Marker 79 | | |  | 0.295 | | |  |  | 0.254 | |  | 1.714 | | |
| Marker 80 | | |  | 0.031 | | |  |  | 0.032 | |  | 0.681 | | |
| Marker 81 | | |  | 0.033 | | |  |  | 0.012 | |  | 0.332 | | |
| Marker 82 | | |  | 0.146 | | |  |  | 0.142 | |  | 1.541 | | |
| Marker 83 | | |  | 0.226 | | |  |  | 0.061 | |  | 0.053 | | |
| Marker 84 | | |  | 0.226 | | |  |  | 0.061 | |  | 0.053 | | |
| Marker 85 | | |  | 0.002 | | |  |  | 0.086 | |  | 0.260 | | |
| Marker 86 | | |  | 0.863 | | |  |  | 1.032 | |  | 0.434 | | |
| Marker 87 | |  |  | 1.630 | |  |  |  | 1.366 | |  | 1.365 | | |
| Marker 88 | |  |  | 2.600 | |  |  |  | 2.553 | |  | 1.894 | | |
|  | Marker 89 |  |  |  | 4.040 |  |  |  |  | 3.820 |  |  | 3.826 |  |
| Marker 92 | |  |  | 0.807 | |  |  |  | 0.873 | |  | 0.747 | | |
| Marker 93 | |  |  | 0.342 | |  |  |  | 0.407 | |  | 0.238 | | |
| Marker 94 | |  |  | 0.172 | |  |  |  | 0.065 | |  | 0.034 | | |
| Marker 95 | |  |  | 0.507 | |  |  |  | 0.410 | |  | 0.504 | | |
| Marker 96 | |  |  | 0.216 | |  |  |  | 0.109 | |  | 0.008 | | |
| Marker 97 | |  |  | 1.805 | |  |  |  | 1.855 | |  | 0.320 | | |
| Marker 98 | |  |  | 2.599 | |  |  |  | 2.397 | |  | 1.456 | | |
| Marker 99 | |  |  | 2.322 | |  |  |  | 2.294 | |  | 2.007 | | |
| Marker100 | |  |  | 2.331 | |  |  |  | 2.433 | |  | 0.981 | | |
| Marker101 | |  |  | 1.811 | |  |  |  | 1.993 | |  | 1.342 | | |
